# Neural speech encoding advantages associated with higher socioeconomic status extend to noise conditions with differential susceptibility

**DOI:** 10.3389/fpsyg.2026.1760305

**Published:** 2026-03-09

**Authors:** Anthony Marcotti, Alejandro Ianiszewski, Vladimir López

**Affiliations:** 1 Escuela de Psicología, Facultad de Ciencias Sociales, Pontificia Universidad Católica de Chile, Santiago, Chile; 2 Escuela de Fonoaudiología, Facultad de Ciencias de la Rehabilitación y Calidad de Vida, Universidad San Sebastián, Santiago, Chile; 3 Centro Interdisciplinario de Neurociencia, Pontificia Universidad Católica de Chile, Santiago, Chile

**Keywords:** frequency-following response, noise susceptibility, socioeconomic status, speech encoding, speech perception in noise

## Abstract

**Introduction:**

Speech perception in noise (SPiN) relies on precise neural encoding of periodic speech cues, which can be assessed using the frequency-following response (FFR). The robustness and fidelity of this encoding vary with maturation, environmental factors, and life experiences. Socioeconomic status (SES), a major contextual determinant of these influences, has been associated with more consistent and higher-quality FFRs in higher-SES individuals. However, it remains unclear whether SES-related advantages in quiet extend to noise. The primary aim was to determine whether SES predicts susceptibility to noise-related degradation in neural encoding, and a secondary aim was to examine whether SES-linked neural differences correspond to behavioral or self-reported SPiN performance.

**Materials and methods:**

Seventy higher-education students with normal hearing were classified into low- and high-SES groups based on maternal education. Speech-evoked FFRs to a 170-ms synthetic /da/ were recorded in quiet and in +10 dB SNR babble. Neural timing, magnitude, and fidelity measures were analyzed. Behavioral SPiN was assessed using a monosyllabic adaptive speech-recognition-threshold task, and self-reported SPiN with the SSQ12. Linear mixed-effects models were used to examine SES effects and their modulation by noise on FFR parameters, and ordinary least-squares regressions were used to test whether these FFR metrics predicted behavioral and self-reported SPiN performance.

**Results:**

Significant interactions between SES and noise indicated differential neural susceptibility to degradation, with higher-SES participants showing smaller noise-related delays in onset and transition timing and reduced declines in fidelity. Larger response magnitudes were also observed in the higher-SES group across segments. Behavioral SPiN showed no consistent group differences, although onset-latency and stimulus-to-response correlation predicted performance. No significant associations were detected for self-reported SPiN.

**Discussion:**

Neural findings indicate that socioeconomic background shapes long-term susceptibility to noise, with higher-SES individuals exhibiting smaller timing delays in both onset and mid-syllabic encoding and more preserved neural fidelity. These advantages may arise from differences in subcortical and cortical phase-locked activity, reflecting neural patterns shaped over development. Maternal education may serve as a proxy for early-life conditions shaped by environmental factors and life experiences during sensitive periods when neural encoding is highly malleable, leaving durable imprints into adulthood.

## 1 Introduction

Speech sounds are acoustically complex, combining rapid spectrotemporal transitions with slower steady-state portions, and require precise neural encoding for robust comprehension in diverse listening environments (Kilgard and Engineer, 2022). This ability, which is central to speech perception in noise (SPiN), depends largely on the encoding of periodic cues such as the fundamental frequency (F_0_) and its harmonics (Stickney et al., 2007; Sayles and Winter, 2008; Chandrasekaran et al., 2009; de Cheveigné, 2021). The frequency-following response (FFR), a phase-locked neural potential evoked by periodic stimuli, provides a reliable electrophysiological correlate of this encoding. Capturing the synchronous activity of neural populations with high temporal and spectral precision (Anderson and Kraus, 2010; Chandrasekaran and Kraus, 2010; Song et al., 2011), FFR metrics have been linked to both self-reported listening abilities and behavioral performance on psychoacoustic SPiN tasks (Parbery-Clark et al., 2009a; Anderson et al., 2010, 2013a, 2013b; Song et al., 2011; Thompson et al., 2019; Bidelman and Momtaz, 2021; Skoe and Kraus, 2024).

As with other electrophysiological responses, FFR parameters and components change across the lifespan due to maturation and aging processes (Skoe et al., 2015). However, environmental factors and life experiences are also key drivers of neural encoding precision. This plasticity is illustrated by populations exposed to distinct linguistic, musical, and physical training experiences. Native Mandarin speakers, exposed from infancy to tonal contrasts, show more precise F_0_ phase locking, stronger harmonic representation, and greater trial-to-trial consistency than English speakers (Krishnan et al., 2005, 2009b; Krishnan et al., 2009a). Musicians, through formal music training, show shorter latencies, larger FFR amplitudes, and improved tracking of rapid F_0_ contours compared with non-musicians (Musacchia et al., 2007; Wong et al., 2007). Athletes, likely benefiting from superior overall health and physical conditioning, show enhanced trial-to-trial consistency and stronger harmonic representation compared with non-athletes (Krizman et al., 2022).

Evidence indicates that stronger FFRs in quiet are associated with less degradation in noise. Parbery-Clark et al. (2009a) found that background noise degraded FFR latencies and harmonic encoding in both musicians and non-musicians, but the reduction was significantly smaller in musicians, reflecting an interaction driven by musicians’ baseline neural advantages. By contrast, Krishnan et al. (2019) reported that reverberation degraded FFR F_0_ amplitude in both native Mandarin and English speakers, but the magnitude of this degradation did not differ between groups despite clear main effects. Thus, even though native Mandarin speakers showed stronger FFRs in quiet, their baseline F_0_ encoding advantage did not translate into smaller susceptibility to degradation under reverberant conditions. Reverberation and noise differ acoustically yet both create adverse listening environments that degrade neural responses. Taken together, these findings suggest that group-related advantages in baseline FFR encoding do not uniformly confer protection in challenging conditions, highlighting the need to examine how environmental factors and life experiences shape not only neural encoding strength but also its susceptibility to degradation.

In addition to variability in susceptibility to degradation, recent evidence indicates that even baseline experiential differences in FFR encoding under adverse listening conditions may not always be robust or consistently observed. In a large-scale multi-site study, Whiteford et al. (2025) found no association between musical training and FFR baseline encoding in background noise, including no enhancement of F_0_ or harmonic representation, and no advantage in stimulus-to-response correlation measures. A separate paradigm assessing dynamic F_0_ tracking in quiet likewise showed no musician-related enhancement. The authors suggested that the discrepancy with earlier studies reporting musician-related enhancements may be attributed to methodological differences, but also to characteristics of the participant samples. Together with the mixed findings regarding susceptibility to degradation (Parbery-Clark et al., 2009a; Krishnan et al., 2019), these results indicate that experiential influences on FFR measures are not uniform and may depend on broader contextual and population-related factors that shape auditory neural function.

Among these broader contextual and population-level influences on environmental factors and life experiences, socioeconomic status (SES) stands out as a major structural determinant. Typically indexed by income, education, and occupation, it is widely recognized as a key determinant of developmental, behavioral, and health outcomes across the lifespan (American Psychological Association, 2007). The impact of SES on neural and behavioral functioning has been widely studied. A comprehensive review by Farah (2017) identified multiple neurocognitive, neurophysiological, and neuroanatomical correlates of SES. For instance, individuals from lower-SES backgrounds exhibit poorer executive-function and related cognitive performance, diminished event related potentials amplitudes, and reduced cortical volume, surface area, and thickness. These differences are thought to arise from reduced access to educational and linguistic stimulation (Fernald et al., 2013), greater exposure to chronic stressors and environmental adversity (Evans and Kim, 2013), and disparities in nutrition and healthcare that affect brain development (Luby et al., 2013).

As expected, SES has also been associated with differences in the FFR. Skoe et al. (2013) studied a group of adolescents whose SES was operationalized based on maternal education. Participants whose mothers’ educational attainment did not extend beyond secondary education were classified as low SES, whereas those whose mothers had pursued higher education were classified as high SES. Adolescents from the low-SES group exhibited less consistent FFRs, reflected in lower stimulus–response correlations, reduced first formant encoding, and noisier spontaneous neural activity compared with their high-SES peers. Similarly, Anderson et al. (2013b) studied middle-aged and older adults and found that a “life experiences” factor, including SES, physical activity, and intellectual engagement, indirectly influenced behavioral SPiN performance via its effect on the FFR, specifically on F_0_, second harmonic, and first formant magnitude encoding, as well as quiet-to-noise correlation (quantifying the similarity between neural responses in noise and quiet). Within this factor, SES, indexed by maternal and self-education, carried the greatest weight, indicating that higher SES was associated with more robust FFR responses and suggesting a substantial cumulative influence of socioeconomic conditions on auditory neural encoding.

The findings of Skoe et al. (2013) and Anderson et al. (2013b) demonstrated that individuals from higher SES backgrounds exhibit more robust neural speech encoding than their lower SES peers. However, it remains unclear whether this advantage confers resistance to noise-related degradation, similar to the pattern observed in musicians, or whether it follows a more uniform degradation pattern, as reported for native Mandarin speakers. Understanding how baseline encoding advantages translate to noisy conditions is particularly important because FFRs measured in noise appear to be even stronger predictors of behavioral SPiN performance than those measured in quiet. In Anderson et al.’s (2013b) study, this was captured by the quiet-to-noise correlation. Within their structural equation model, this measure showed a strong loading onto a latent Central Processing factor, alongside pitch encoding and first-formant encoding obtained in quiet, which were also included as neural indicators of that construct. Central Processing, in turn, directly predicted SPiN ability, represented as a latent variable composed of three psychoacoustic SPiN tasks. Notably, in *post hoc* regression analyses using the psychoacoustic SPiN test with the highest loading on that latent variable, the quiet-to-noise correlation remained one of the strongest predictors alongside auditory working memory, whereas the FFR metrics obtained in quiet were not significant.

If higher SES confers measurable advantages in the neural encoding of speech in noise, these advantages may contribute to disparities in everyday listening success across communication, education, and the workplace. SES-linked differences in FFRs, particularly under noisy conditions, would therefore provide insight into the neural bases of SPiN performance and highlight socioeconomic disadvantage as a factor shaping access to effective communication. Establishing FFRs in noise as potential markers of these SES-related differences could also inform the design of interventions aimed at mitigating their impact, for example through targeted auditory training or environmental modifications. Accordingly, the primary aim of the present study was to determine whether SES is associated with differential susceptibility to noise-related degradation in neural speech encoding. The secondary aim was to examine whether SES-linked differential effects of noise on neural responses are mirrored by corresponding SES-related differences in behavioral and self-reported SPiN performance.

## 2 Materials and methods

All procedures were conducted at the Audiology Laboratory of the School of Speech-Language Pathology at Universidad San Sebastián, Los Leones Campus, located in the Metropolitan Region of Chile. The study protocol was approved by the Scientific Ethics Committee for Social Sciences, Arts, and Humanities of the Pontificia Universidad Católica de Chile (ID 240424011) and was conducted in accordance with the Declaration of Helsinki and its subsequent amendments. All participants provided written informed consent and received financial compensation for their participation.

### 2.1 Participants

A total of 70 Spanish-speaking Chilean participants aged between 18 and 30 years were assessed, all of whom were higher-education students residing in the Metropolitan Region of Chile. Participants were categorized into low- and high-SES groups based on maternal education, as reported by the participant. Maternal education was originally collected using detailed categories (primary education, secondary education, technical or professional training, university education, and postgraduate studies). For SES classification, these categories were collapsed into a dichotomous variable reflecting whether the mother had completed tertiary education by the time the participant was 12 years old. Participants whose mothers had not completed tertiary education by the time the participant reached 12 years of age were assigned to the low-SES group, whereas those whose mothers had completed tertiary education within that period were assigned to the high-SES group. Independently of this grouping criterion, the detailed of maternal education were also recorded and are reported descriptively to provide a more comprehensive characterization of the sample. The 12-year cutoff was chosen because the speech-evoked FFR shows marked developmental changes throughout childhood, including a pronounced overshoot between approximately 5 and 11 years, before stabilizing near early adolescence, a period characterized by rapid developmental change and heightened sensitivity to environmental input (Skoe et al., 2015). This makes maternal education particularly relevant during these years, when auditory neural encoding is most malleable. Maternal education was selected as the SES indicator because it is a well-established predictor of early developmental environments, capturing differences in access to resources, health-seeking behavior, and linguistic and cognitive stimulation in the home, with long-term consequences that extend into adolescence and adulthood (American Psychological Association, 2007).

Participants were included if they had no formal musical training beyond the mandatory courses in the official Chilean primary and secondary education curriculum and did not report bilingual-level proficiency in English or any other foreign language. They also had no history or current diagnosis of neurodevelopmental disorders such as attention-deficit/hyperactivity disorder, language development disorders, or autism spectrum disorder, nor recent hearing pathology or recurrent otological conditions during childhood or developmental years. In addition, all participants were required to have normal hearing, confirmed by clinical audiometry with pure-tone thresholds ≤20 dB HL between 125 and 8,000 Hz, a speech recognition score ≥92% in quiet, bilateral type A tympanograms, and the presence of both ipsilateral and contralateral acoustic reflexes at 500, 1,000, and 2,000 Hz.

In addition to SES grouping, the sample was further characterized using demographic and socioeconomic variables, including age, sex, handedness, type of primary and secondary school attended, type of higher education institution, paternal education, family income in Chilean pesos (CLP), number of household members, and health coverage. In Chile, school education is provided through three types of institutions: public schools, which are fully state-funded; subsidized private schools which are privately administered but partially state-funded; and private schools, which are fully funded through tuition fees. When participants had attended more than one type of school, classification was based on the type in which they had spent the greatest number of years. Higher education is also structured into three types of institutions: technical training centers, which focus on vocational education; professional institutes, which offer technical and professional programs; and universities, which grant academic and professional degrees. The Chilean health system comprises FONASA, which is a public and free health coverage system, ISAPRE, which consists of private health insurance providers requiring monthly contributions, and other specific schemes providing free coverage for members of the Armed Forces, Police, and their families.

### 2.2 Sample size estimation

Sample size was calculated using a Monte Carlo simulation–based power analysis (10,000 iterations) for linear mixed-effects models, as analytical solutions are generally not available for designs that include both fixed and random effects. Simulation-based approaches are recommended because power can be estimated by repeatedly generating data under a prespecified model and computing the proportion of significant effects (Kumle et al., 2021). The simulated model mirrored the planned analysis (see Statistical Analysis section) and was specified to allow adjustment for up to two subject-level covariates if required. The primary effect of interest was the interaction between SES and noise condition. Interaction effect parameters were derived from Parbery-Clark et al. (2009a). To ensure a conservative power estimate, variance components for both subject-level random effects and residual error were specified at levels reflecting moderate-to-high variability, and the simulation explicitly allowed for the inclusion of up to two covariates. Statistical inference was based on a Likelihood Ratio Test (LRT) comparing models with and without the interaction term (Wiley and Rapp, 2019). Power was defined as the proportion of simulations yielding *p* < 0.05. Simulations indicated that power exceeded 0.80 with 64 participants. To account for potential data loss and reductions in effective sample size at the participant level, the planned sample was conservatively increased by six additional participants, resulting in a final target sample of 70 subjects.

### 2.3 Stimulus and recording

First, auditory brainstem responses (ABRs) were obtained using a conventional click stimulus presented monaurally at 80 dB SPL to confirm the integrity of the auditory pathway at the brainstem level. Then, FFR were elicited using a synthetic 170-ms /da/ syllable generated with a Klatt synthesizer at a 20 kHz sampling rate (Klatt, 1980). The stimulus is available for download from the Auditory Neuroscience Laboratory “Brainvolts” at Northwestern University.^1^ The stimulus comprises three temporal segments: onset, transition, and steady-state (Anderson et al., 2012). The onset corresponds to the initial 5 ms and includes the consonant stop burst of the /d/. After this burst, voicing remains constant with a F_0_ of 100 Hz throughout the following two temporal segments. During the transition segment (20–60 ms), as the syllable moves from the /d/ to the /a/, the lower three formants shift in frequency: F1 from 400 to 720 Hz, F2 from 1,700 to 1,240 Hz, and F3 from 2,580 to 2,500 Hz. These formants then stabilize during the steady-state vowel segment (60–170 ms). The fourth through sixth formants (F4-F6) remain constant across the transition and steady-state segments at 3300, 3750, and 4,900 Hz, respectively. This syllable was presented monaurally at 80 dB SPL under two listening conditions: in quiet, and in the presence of six-talker babble background noise (three male and three female voices) at a + 10 dB signal-to-noise ratio (SNR), with the babble presented at 70 dB SPL.

All responses were recorded using the SmartEP v5.54 module of the Duet system (Intelligent Hearing Systems, Miami, FL). A vertical electrode montage with four Ag-AgCl electrodes was used, with the active electrode placed at Cz, ground at Fpz, and references at M1 and M2. Electrode impedances were kept below 3 kΩ, with inter-electrode impedance differences below 1.5 kΩ. Stimuli for both ABR and FFR recordings were presented monaurally in alternating polarity through electromagnetically shielded insert earphones. Common acquisition parameters included an online band-pass filter from 70 to 3,000 Hz, artifact rejection for activity exceeding ±32 μV, and a sampling rate of 13.33 kHz. For the ABR, responses were obtained only in quiet, using 2,048 sweeps and a stimulation rate of 21.1 Hz. For the FFR, two trials of 2,048 sweeps per ear were collected for each condition (quiet and noise), using a stimulation rate of 4.35 Hz. All acquisition parameters followed current methodological recommendations for brainstem auditory evoked potentials and FFR recordings (Skoe and Kraus, 2010).

### 2.4 Data reduction

All waveforms were baseline-corrected using the pre-stimulus interval (−40 to 0 ms) prior to subsequent analyses. The two polarities of each waveform were then added to minimize the influence of cochlear microphonic and stimulus artifact on the response, as these components invert with stimulus polarity and are thus attenuated by summation (Krizman and Kraus, 2019). Within each ear, the resulting added waveforms from the two trials were then averaged. Finally, left and right ear averages were combined to obtain a single waveform per condition. The resulting waveforms were digitally band-pass filtered offline using a zero-phase forward-backward Butterworth filter with cutoff frequencies of 70 Hz (high-pass) and 2,000 Hz (low-pass), selected to retain spectral components relevant to FFR phase-locked activity while attenuating low-frequency cortical contributions and high-frequency noise (Skoe and Kraus, 2010). The second-order filter, applied in both forward and reverse directions, yielded an effective fourth-order response with an attenuation slope of approximately 24 dB per octave at each cutoff.

### 2.5 ABR analyses

For the ABR, analyses focused on two sets of measures. Absolute latencies were identified for waves I, III, and V, while inter-peak latencies were calculated for I-III, III-V, and I-V. Peak-to-peak amplitudes were also measured for waves I, III, and V, defined as the distance between the most positive and the most negative deflection within the canonical latency window of each wave. Peak identification was performed using predefined latency windows based on established norms, and all peaks were visually inspected and confirmed by a trained member of the research team.

### 2.6 FFR analyses

All FFR preprocessing and analyses were performed in MATLAB R2025b (The MathWorks Inc., 2025) using custom scripts developed for this study. Filtering, windowing and other signal-processing steps used functions from the Signal Processing Toolbox, while all additional operations relied on native MATLAB functions.

#### 2.6.1 Neural timing

Typical positive peaks evoked by the 170-ms /da/ syllable were identified and labeled based on their expected latencies (e.g., a peak occurring 61–63 ms after stimulus onset was labeled “peak 62”). Peak identification was performed using a custom automated peak-detection algorithm developed in MATLAB, which detected local maxima and minima within predefined time windows. These windows were set according to expected latencies reported in previous studies (Anderson et al., 2012; Krizman and Kraus, 2019). All automatically detected peaks were subsequently reviewed and manually adjusted by a trained member of the research team with prior experience in analyzing FFR waveforms. The onset response was identified as peak 9. The transition segment included peaks 23, 32, 42, and 52, while the steady-state segment comprised peaks 62 through 162.

#### 2.6.2 Magnitude

Two types of magnitude measures were analyzed: broadband and frequency-specific. Broadband magnitude was estimated by calculating the root mean square (RMS) of the FFR waveform. Frequency-specific magnitude was derived using the fast Fourier transform (FFT), focused on the fundamental frequency (F_0_ = 100 Hz) and its first two harmonics (H_2_ = 200 Hz, H_3_ = 300 Hz). To enhance spectral precision, zero-padding was applied to match the number of points in the FFT to the sampling rate, yielding a spectral resolution of 1 Hz/bin. This approach ensured that spectral components aligned precisely with integer frequency bins, thereby improving measurement accuracy. Mean spectral amplitudes were computed by averaging across 20-Hz windows centered on each frequency of interest, consistent with previous studies (Anderson et al., 2012). RMS magnitude was computed for all segments: pre-stimulus (−40–0 ms), transition (20–60 ms), steady-state (60–170 ms), and full-stimulus (5–180 ms). FFT-based spectral analyses were performed only in the steady-state segment (60–170 ms).

#### 2.6.3 Fidelity

Two types of fidelity measures were analyzed: stimulus-to-response correlation and response consistency correlation. The stimulus-to-response correlation refers to the morphological similarity between the FFR waveform and the evoking stimulus, in this case the syllable /da/. To obtain this measure, the stimulus was processed with the same zero-phase forward-backward Butterworth filter that was applied to the FFR responses. The response consistency correlation or response-to-response correlation corresponds to the within-session reliability of FFR recordings, calculated as the correlation between two waveforms. For this measure, two independent waveforms per condition were specifically constructed by averaging corresponding trials across ears (left trial 1 + right trial 1; left trial 2 + right trial 2), resulting in 4096 sweeps per waveform (2,048 from each ear). Both stimulus-to-response and response consistency correlations were computed within three time segments: the transition (20–60 ms), the steady-state (60–170 ms), and the full-stimulus window (5–180 ms). Correlation values were Fisher-transformed to z-scores prior to statistical analyses.

### 2.7 Behavioral SPiN performance

An experimental psychoacoustic SPiN test was developed to estimate the speech recognition threshold (SRT), defined as the lowest SNR at which a listener can correctly recognize at least 50% of the presented words. A list of 200 monosyllabic words was compiled from existing Spanish logoaudiometric materials and supplemented with commonly used everyday words. The words were recorded by a female speaker in neutral Spanish and presented monaurally in continuous six-talker babble, the same noise used in the FFR noise condition. The babble was fixed at 30 dB above the participant’s average pure-tone thresholds across 500–4,000 Hz. Stimuli were delivered through an adaptive staircase procedure in which the SNR decreased by 2 dB following correct responses and increased by 2 dB following incorrect responses. Each ear was tested independently for up to 30 trials, beginning at an SNR of +10 dB. The SRT for each ear was estimated as the mean SNR of the final six reversals, which capture the stable convergent region of the staircase where transformed up-down methods yield robust, low-bias threshold estimates (Levitt, 1971).

This approach addresses key limitations of Spanish SPiN tests such as the Speech-in-Noise (SIN) subtest of the Santiago APD battery, the Hearing in Noise Test (HINT), and QuickSIN. The SIN test uses only two fixed SNRs and often produces ceiling effects in young adults with normal hearing (Fuente and McPherson, 2006), limiting its sensitivity. In contrast, HINT (Nilsson et al., 1994) and QuickSIN (Killion et al., 2004) employ semantically predictable sentences, which is problematic given that SES influences linguistic experience and semantic knowledge (van der Kleij et al., 2023), potentially confounding results (Braza et al., 2022). Performance on these sentence-based tests also depends on working memory (Ingvalson et al., 2015) and SPiN ability itself has been independently linked to working memory (Lad et al., 2020), a domain where SES-related differences have been reported (Evans and Schamberg, 2009). By using monosyllabic words with minimal contextual predictability, the present design reduces the influence of vocabulary and working memory, enabling a more direct assessment of auditory processing in noise.

### 2.8 Self-reported SPiN performance

For the measurement of self-reported SPiN, the Spanish version of the SSQ12, validated in the Chilean adult population, was used (Cañete et al., 2022). This instrument is a 12-item version of the Speech, Spatial and Qualities of Hearing Scale (SSQ49) designed for rapid assessment in clinical and research settings (Noble et al., 2013). Each item represents a common everyday listening situation and is organized into one of three main domains: speech perception, spatial hearing, and hearing qualities. Items are rated on a scale from 0 to 10, with scores close to 10 indicating that the task can be performed without difficulty and scores close to 0 indicating great difficulty or inability to perform it.

### 2.9 Lexical knowledge and working memory

Because both lexical knowledge and working memory are key determinants of speech-in-noise perception and show significant variation across socioeconomic levels, these abilities were assessed using subtests from the Chilean standardized version of the Wechsler Adult Intelligence Scale-Fourth Edition (WAIS-IV) (Rosas et al., 2014). Lexical knowledge was measured with the Vocabulary subtest, in which participants define orally presented words, and responses are scored for accuracy and quality. The total raw score, obtained by summing the item scores, was used for statistical analyses, with higher values reflecting greater and more precise lexical knowledge. Working memory was evaluated with a composite index derived from the forward digit span, which involves repeating number sequences in the order presented; the backward digit span, which requires repeating them in reverse order; and the sequencing digit span, which requires repeating them in ascending numerical order. Raw scores from these three subtests were summed to create the working memory measure used in the analyses, with higher values indicating better working memory performance.

### 2.10 Statistical analysis

Medians with interquartile ranges (p25-p75) were reported for numerical variables, and absolute and relative frequencies for categorical variables. Between-group comparisons were performed using the Wilcoxon rank-sum test for numerical variables, with the Z statistic reported, and Fisher’s exact test for categorical variables, with the *χ*^2^ statistic reported. Correlation analyses were conducted using Pearson’s correlation coefficient (*ρ*) to examine associations between cognitive measures (lexical knowledge and working memory), FFR parameters, behavioral SPiN performance, and self-reported SPiN.

For the ABR timing analysis, two separate sets of linear mixed-effects models (LMMs) were fitted: one for wave latencies (I, III, and V) and another for interpeak intervals (I–III, III–V, and I–V). In both models, SES and wave/interpeak type were entered as fixed effects, while subject was modeled as a random intercept to account for repeated measures. Peak-to-peak amplitudes (waves I, III, and V) were analyzed using LMMs with SES as fixed effect and subject as random intercept.

For the FFR timing analysis, LMMs were fitted for each response segment. Fixed effects included SES and noise condition; for the transition and steady-state segments, peak number was also included as a numeric variable. For the analysis of RMS, F_0_, H_2_, and H_3_, LMMs were fitted to log-transformed values. The logarithmic transformation addressed the positively skewed distribution of the original measures, strictly positive values with long right tails, thus improving adherence to assumptions of normality and homoscedasticity. Stimulus-to-response and response-to-response correlations were also examined using LMMs. All LMMs included random intercepts for subject and were estimated using restricted maximum likelihood (REML), given its robustness for mixed-effects estimation with small samples (McNeish, 2017). For models including SES and noise condition as predictors, an additional specification with the interaction between SES and noise condition was estimated to assess potential moderation. Model selection followed a parsimony criterion: when the interaction was not significant, the main-effects model was retained. This hierarchical selection strategy limited model multiplicity by ensuring that inference was based on a single retained specification rather than parallel interpretation of alternative models, and it is consistent with statistical principles favoring parsimonious nested models that yield more precise and reliable estimates (Bentler and Mooijaart, 1989).

For the behavioral and self-reported SPiN performance analyses, ordinary least-squares (OLS) linear models regressions were specified using SES and FFR-derived contrast metrics, defined as the silence-babble difference, as predictors. Regression models were estimated only for those FFR parameters that had shown a statistically significant interaction between SES and noise condition in the preceding analyses, because these parameters yielded contrast metrics that validly indexed SES-related differential neural susceptibility to noise and were therefore appropriate for examining associations with behavioral and self-report outcomes. A hierarchical regression framework was implemented, where an initial SES-only model was followed by an additive model including the FFR contrast, and finally by an interaction model that incorporated the interaction between SES and the FFR contrast to assess whether the association between the neural contrast and SPiN performance differed by SES.

Sensitivity analyses were conducted by replicating all regressions after excluding outliers, which were identified within each SES-by-condition group using the interquartile range (IQR) method. Specifically, values lower than the first quartile minus 1.5 times the IQR or higher than the third quartile plus 1.5 times the IQR were flagged as outliers. These models were prespecified solely to evaluate the robustness of the primary findings and were not considered an alternative basis for inference. Accordingly, interpretation in the main text is restricted to the primary models, whereas results from sensitivity analyses are reported in the Supplementary material. Together with the hierarchical and parsimonious model-selection strategy described above, this approach provided an additional safeguard against spurious findings by verifying that the substantive conclusions did not depend on extreme observations or alternative model specifications.

To obtain inference that does not rely on distributional assumptions, all mixed-effects models were re-estimated using non-parametric cluster bootstrapping with 10,000 replications, resampling at the participant level (Field and Welsh, 2007). OLS linear models regressions were estimated using non-parametric bootstrapping with 10,000 replications in combination with HC3 robust standard errors, which provide reliable inference under potential heteroscedasticity and small sample sizes (Long and Ervin, 2000). All statistical analyses were conducted in R version 4.5 (R Core Team, 2025).

## 3 Results

According to maternal education, 14.3% (*n* = 5) of Low-SES participants had mothers with primary education and 85.7% (*n* = 30) with secondary education. In contrast, the High-SES group included 37.1% (*n* = 13) with maternal technical/professional training, 54.3% (*n* = 19) with university education, and 8.6% (*n* = 3) with postgraduate studies. Beyond maternal education, significant group differences were also observed in paternal education (*χ*^2^ = 30.291, *p* < 0.001), family income (Z = −2.768, *p* = 0.005), and health coverage (*χ*^2^ = 16.845, *p* < 0.001). Participants in the High-SES group more frequently reported fathers with higher educational attainment, particularly university education (38.24%), with secondary (23.53%) and postgraduate education (23.53%) equally represented. In contrast, the Low-SES group more commonly reported secondary education (73.53%), followed by primary education (14.71%) and university education (5.88%). Median family income was higher in the High-SES group (1,800,000 CLP [950,000–3,500,000]) compared with the Low-SES group (1,060,000 CLP [735,000–1,400,000]), and private health coverage (ISAPRE) was more frequent in the High-SES group (51.43%), whereas public coverage (FONASA) predominated in the Low-SES group (82.86%). No significant differences were observed in other sociodemographic variables. Further details of these descriptive and comparative statistics are provided in Table 1.

**Table 1:** Descriptive and comparative statistics for low-SES and high-SES groups.

Variable	Low-SES	High-SES	Test statistic	*p*-value
Age	22 (20.00; 25.00)	22.00 (21.00; 25.00)	0.136	0.895
Sex				
Male	14 (40.00)	12 (34.29)	0.402	0.621
Female	21 (60.00)	23 (65.71)		
Handedness				
Right	30 (85.71)	33 (94.29)	1.429	0.214
Left	5 (14.29)	2 (5.71)		
Education establishment				
Technical training center	1 (2.86)	–	3.024	0.188
Professional institute	7 (20.00)	3 (8.57)		
University	27 (77.14)	32 (91.43)		
Paternal education				
No response	1 (2.94)	1 (2.94)	30.291	<0.001
No schooling	1 (2.94)	–		
Primary	5 (14.71)	1 (2.94)		
Secondary	25 (73.53)	8 (23.53)		
Technical	1 (2.94)	4 (11.76)		
University	2 (5.88)	13 (38.24)		
Postgraduate	–	8 (23.53)		
Family income	1,060,000 (735,000; 1,400,000)	1,800,000 (950,000; 3,500,000)	−2.768	0.005
Household members	3 (2.00; 4.00)	3 (1.00; 4.00)	7.750	0.418
Health coverage				
FONASA	29 (82.86)	17 (48.57)	16.845	<0.001
ISAPRE	3 (8.57)	18 (51.43)		
Other	3 (8.57)	–		
Vocabulary	26.00 (19.00–32.00)	32.00 (23.00–37.00)	−2.969	0.003
Working memory	25.00 (22.00–28.00)	27.00 (24.00–30.00)	−2.080	0.037

For numeric variables (age, family income, and household members, vocabulary and working memory), values are presented as medians with 25th and 75th percentiles in parentheses. For categorical variables (sex, handedness, education establishment, paternal education, and health coverage), values are presented as absolute counts with percentages in parentheses. Comparisons for numeric variables used the Wilcoxon rank-sum test (*Z* reported), while categorical comparisons used Fisher’s exact test (*χ*^2^ reported). FONASA is Chile’s public health coverage system, ISAPRE denotes private health coverage, and “Other” includes special coverage for the Armed Forces, Police, and related services. Family Income is reported in Chilean pesos.

Significant group differences were also observed in lexical knowledge and working memory. Lexical knowledge scores were lower in the Low-SES group (median = 26.00, IQR = 19.00–32.00) compared with the High-SES group (median = 32.00, IQR = 23.00–37.00; z = −2.969, *p* = 0.003). Working memory scores were also lower in the Low-SES group (median = 25.00, IQR = 22.00–28.00) than in the High-SES group (median = 27.00, IQR = 24.00–30.00; z = −2.080, *p* = 0.037). Correlations were then examined between these cognitive measures and FFR parameters, the psychoacoustic SPiN test, and self-reported SPiN. Lexical knowledge showed no significant associations with any measure. In contrast, working memory correlated with multiple FFR indices. In quiet, significant correlations were found with RMS values (transition, steady-state, full stimulus), amplitudes (F_0_, H_2_), and the stimulus-to-response correlation for the full stimulus. In noise, working memory was again associated with RMS values (transition, steady-state, full stimulus), amplitudes (F_0_, H_2_, H_3_), and stimulus-to-response correlations (full stimulus, steady-state). Working memory was therefore included as a covariate in subsequent models. Full correlation matrices are provided in Supplementary material 1.

### 3.1 Auditory brainstem response

**Figure 1:**
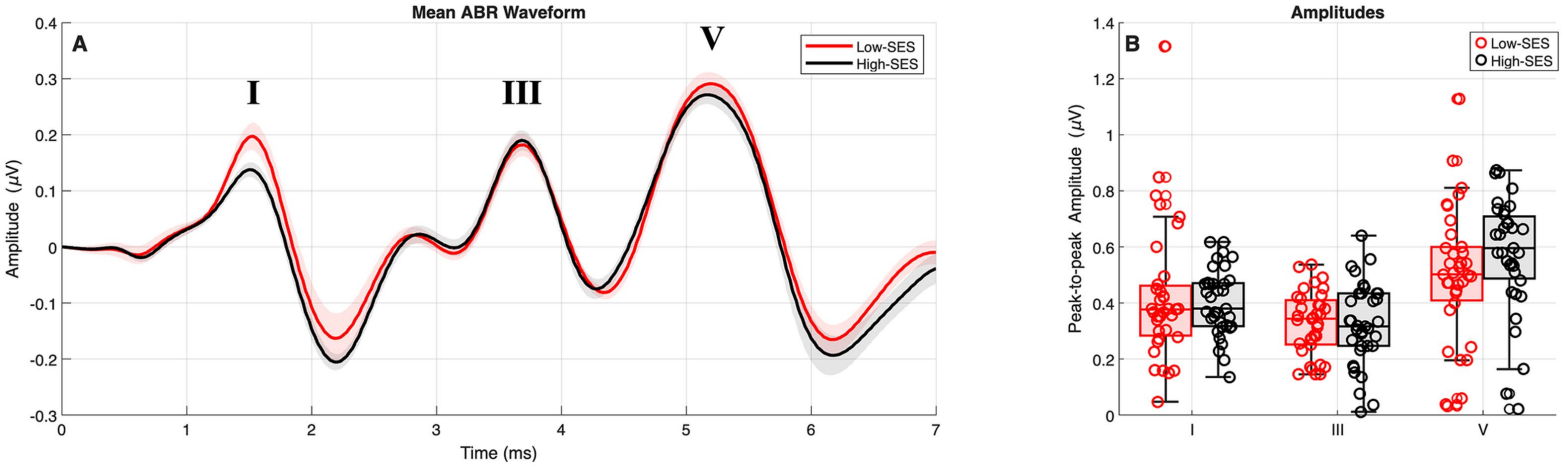
ABR results for High-SES (black) and Low-SES (red) groups. Panel **A** displays the morphology of the averaged waveforms, with broadly comparable absolute latencies (ms) and inter-peak latencies (I–III, III–V, I–V) across groups. Shaded areas represent ±1 standard error of the mean (SEM) across participants within each group. Panel **B** presents the peak-to-peak amplitudes (μV) for waves I, III, and V. The groups exhibited similar latency patterns, while a subtle amplitude difference emerged only after outlier removal, with the High-SES group exhibiting comparatively larger responses. Outliers were not removed for visualization.

No significant main effects of SES were observed for ABR absolute latencies (*β* = −0.040, *p* = 0.211) or inter-peak latencies (*β* = −0.027, *p* = 0.589), indicating no differences between groups. Likewise, no SES effects were detected for peak-to-peak amplitudes (*β* = 0.016, *p* = 0.493). The results of these analyses are shown in Figure 1. Further details of the models and findings from the sensitivity analyses are provided in Supplementary material 2.

### 3.2 Frequency following response

#### 3.2.1 Timing

**Figure 2:**
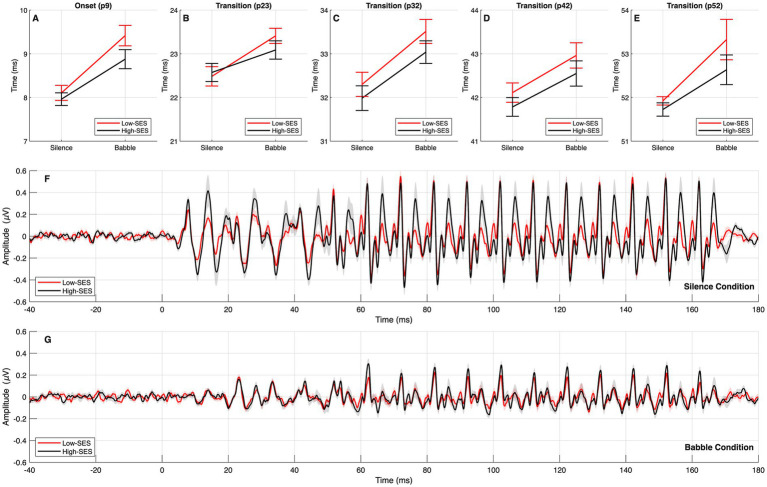
FFR latency results for High-SES (black) and Low-SES (red) groups. Panels **A–E** display latencies for the onset peak (p9) and transition peaks (p23, p32, p42, and p52). Significant SES effects were observed in both the onset and transition segments, reflecting differences in susceptibility to noise. Although noise systematically delayed responses, this effect was less pronounced in the High-SES group. Within the transition segment, an additional SES main effect indicated longer latencies in the Low-SES group. No SES-related differences were found for steady-state peaks. Panels F and G present the average waveform morphology in the silence and babble conditions, respectively, illustrating the changes in morphology and amplitude induced by noise exposure. Shaded areas represent ±1 standard error of the mean (SEM) across participants within each group. Outliers were not removed for visualization.

The median and IQR of observed latencies are provided in Supplementary material 3. Notably, the onset component could not be reliably identified in five subjects in the babble condition, two from the Low-SES group and three from the High-SES group, so the onset-latency analyses were conducted on the remaining 65 participants. In the onset segment, the interaction between SES and noise condition was significant (*β* = −0.402, *p* = 0.008), while the main effect of SES was not (*β* = −0.143, *p* = 0.143), and noise significantly increased latencies (*β* = 1.267, *p* < 0.001). In the transition segment, the interaction was significant (*β* = −0.287, *p* = 0.009), and SES showed an additional main effect (*β* = −0.188, *p* = 0.023), with a significant main effect of noise (*β* = 1.098, *p* < 0.001). In the steady-state segment, the interaction was not significant (*β* = −0.041, *p* = 0.748). The model was therefore re-estimated without the interaction term between SES and noise condition, and this re-estimated model showed no significant main effect of SES (*β* = −0.089, *p* = 0.235), while noise remained a significant predictor of longer latencies (*β* = 0.389, *p* < 0.001). Across models, noise consistently increased latencies (*p* < 0.001), and peak number indicated progressive delays at later peaks (*p* < 0.001). Thus, noise systematically delayed neural responses, with SES-related differences confined to onset and transition. The effect of noise was smaller in the High-SES group. Findings are illustrated in Figure 2. Further details of the models and the sensitivity analyses are provided in Supplementary material 4.

#### 3.2.2 Magnitude

**Figure 3:**
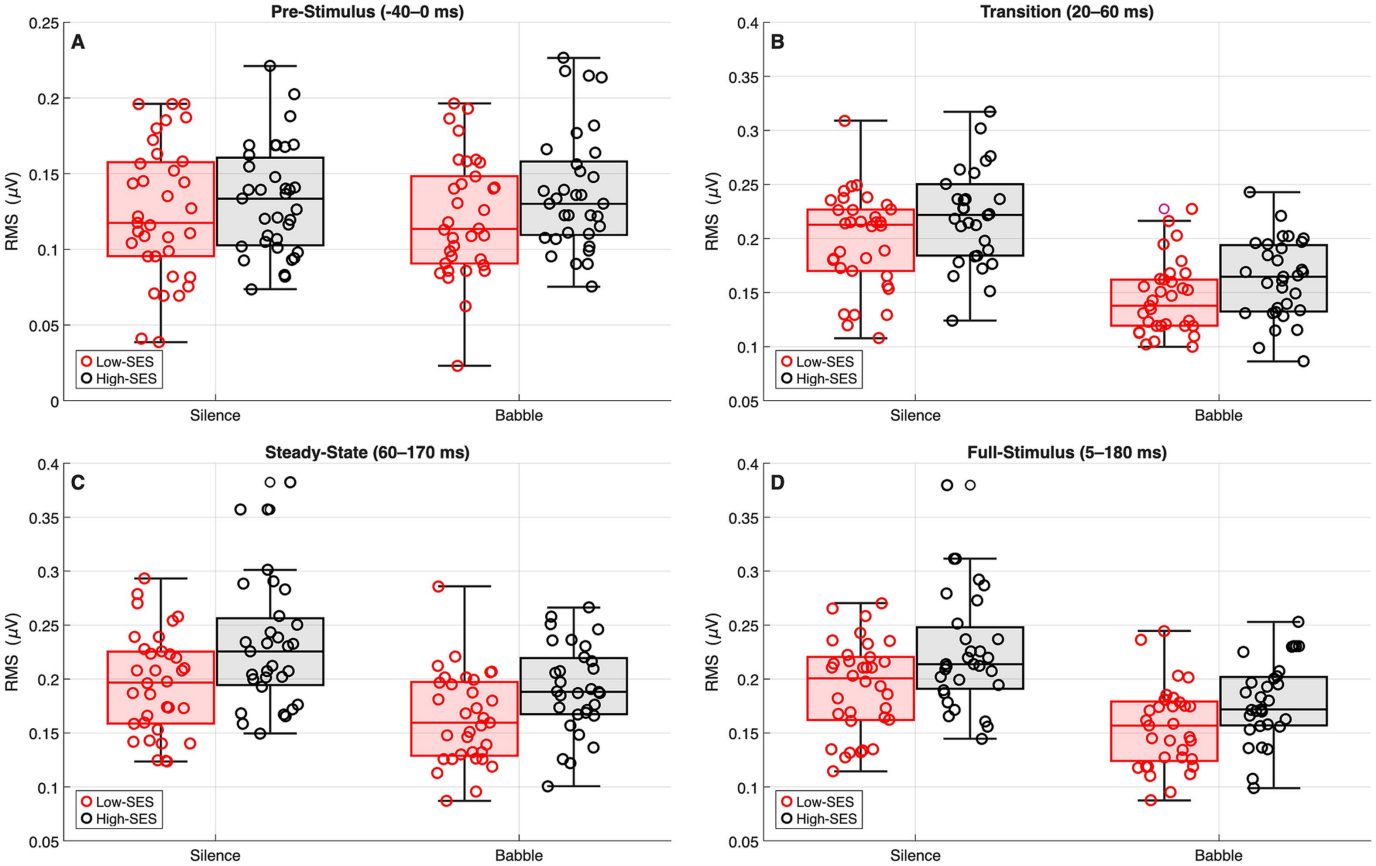
Root mean square (RMS) broadband magnitude values (μV) for High-SES (black) and Low-SES (red) groups in silence and babble conditions. Panels **A–D** correspond to the pre-stimulus (−40–0 ms), transition (20–60 ms), steady-state (60–170 ms), and full-stimulus (5–180 ms) intervals, respectively. Across segments, the High-SES group exhibited consistently larger RMS amplitudes. Noise markedly reduced responses in the transition, steady-state, and full-stimulus intervals, whereas pre-stimulus activity remained comparable across conditions. Outliers were not removed for visualization.

The median and IQR of the observed RMS broadband magnitude values are provided in Supplementary material 5. As the interaction between SES and noise condition was not significant in any segment (*p* > 0.05), models were re-estimated without this interaction term. Across segments, SES consistently predicted larger amplitudes for the High-SES group relative to the Low-SES group: pre-stimulus *β* = 0.136, *p* = 0.022; transition *β* = 0.159, *p* = 0.005; steady-state *β* = 0.207, *p* = 0.002; full-stimulus *β* = 0.195, *p* = 0.002. Noise condition also showed robust effects in the transition (*β* = −0.341, *p* < 0.001), steady-state (*β* = −0.205, *p* < 0.001), and full-stimulus segments (*β* = −0.240, *p* < 0.001), but not in the pre-stimulus segment (*β* = 0.051, *p* = 0.327). Thus, higher SES was associated with consistently larger broadband responses, while noise reliably reduced amplitudes, and these effects occurred independently, as no significant interaction between SES and noise condition was observed in any segment. These effects are illustrated in Figure 3. Further details of the models and the sensitivity analyses are provided in Supplementary material 6.

**Figure 4:**
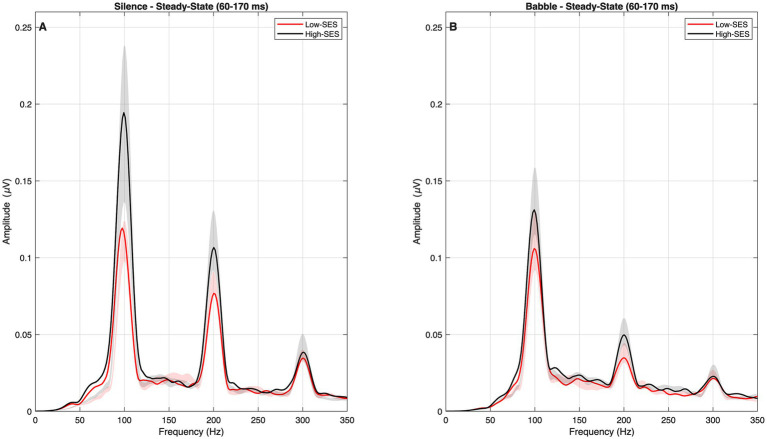
Harmonic-specific response magnitudes (μV) in the steady-state segment (60–170 ms) for High-SES (black) and Low-SES (red) groups. Panel **A** presents results in the silence condition, and Panel **B** presents results in the babble condition. Across panels, background noise produced a clear reduction in harmonic amplitudes. The High-SES group exhibited consistently larger magnitudes across conditions. Shaded areas represent ±1 standard error of the mean (SEM) across participants within each group. Outliers were not removed for visualization.

With respect to the harmonics, the median and IQR of the observed frequency-specific magnitude values are provided in Supplementary material 7. No significant interaction between SES and noise condition was observed (*β* = −0.002, *p* = 0.971). The re-estimated model showed significant main effects of SES (*β* = 0.265, *p* = 0.003) and noise condition (*β* = −0.406, *p* < 0.001). Magnitudes also varied systematically across harmonic components, being lower for the H_2_ (*β* = −0.871, *p* < 0.001) and the H_3_ (*β* = −1.504, *p* < 0.001) compared with the F_0_. Overall, High-SES was consistently associated with larger harmonic amplitudes, while background noise reduced response magnitude, with no significant interaction between SES and noise condition observed across segments. These effects are illustrated in Figure 4. Further details of the models and the sensitivity analyses are provided in Supplementary material 8.

#### 3.2.3 Fidelity

**Figure 5:**
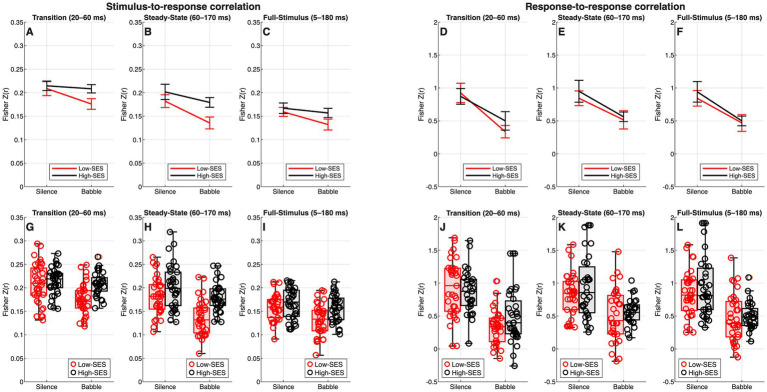
Stimulus-to-response and response-to-response correlations (z-transformed) for High-SES (black) and Low-SES (red) groups in silence and babble conditions. Panels **A–C** show stimulus-to-response correlations in the transition (20–60 ms), steady-state (60–170 ms), and full-stimulus (5–180 ms) intervals. In these segments, background noise reduced correlations, with a smaller reduction in the High-SES group. Panels **D–F** present response-to-response correlations for the same intervals, showing uniform noise-related decreases and no SES effects. Panels **G–I** and **J–L** depict the distribution of individual data points for stimulus-to-response and response-to-response correlations, respectively, across silence and babble conditions. Outliers were not removed for visualization.

The median and IQR of the observed z-score-transformed stimulus-to-response correlation values for all segments are provided in Supplementary material 9. Significant interactions between SES and noise condition were found in the transition (*β* = 0.026, *p* = 0.018), steady-state (*β* = 0.024, *p* = 0.034), and full-stimulus segments (*β* = 0.019, *p* = 0.041). In the transition and full-stimulus, SES alone was not significant (*β* = 0.006, *p* = 0.445; *β* = 0.002, *p* = 0.732, respectively), whereas in the steady-state SES showed a marginal effect (*β* = 0.017, *p* = 0.065). Across models, background noise consistently reduced stimulus-to-response correlations (*p* < 0.01). This effect was smaller in the High-SES group, as reflected in the significant interactions. These effects are illustrated in Figure 5. Full model details and sensitivity analyses are provided in Supplementary material 10.

With respect to the response-to-response correlation, the median and IQR of the observed z-score-transformed values for all segments are provided in Supplementary material 11. As no significant interaction between SES and noise condition was observed in any segment (*p* > 0.05), models were re-estimated without this interaction term. Across segments, SES showed no significant main effects: in the transition (*β* = 0.080, *p* = 0.255), in the steady-state (*β* = 0.132, *p* = 0.082), and in the full-stimulus (*β* = 0.117, *p* = 0.103). By contrast, background noise consistently reduced response-to-response correlations (*p* < 0.001). Overall, these findings indicate that response-to-response correlations were consistently reduced by background noise, with no SES-related differences either as main effects or interactions. These results are illustrated in Figure 5. Full model details and sensitivity analyses are provided in Supplementary material 12.

#### 3.2.4 Behavioral and self-reported SPiN

The median and IQR values for both behavioral SPiN performance and self-reported measures are presented in Supplementary material 13. In these analyses, the FFR predictors included the latency contrasts (onset, transition) and the stimulus-to-response correlation contrasts (transition, steady-state, and full-stimulus), selected on the basis of the significant interactions observed in the preceding models. For behavioral SPiN performance, no significant interactions between SES and any of the FFR contrasts were detected in any of the models; therefore, additive specifications were retained for interpretation. No significant main effects of SES were observed in any model. Regarding the FFR contrast predictors, significant associations were observed for the onset latency contrast (*β* = 1.039, *p* = 0.008) and for the full-stimulus stimulus-to-response correlation contrast (*β* = 15.369, *p* = 0.018). The transition-latency contrast (*β* = −0.018, *p* = 0.883), the transition stimulus-to-response correlation contrast (*β* = 6.725, *p* = 0.082), and the steady-state stimulus-to-response correlation contrast (*β* = 9.381, *p* = 0.053) were not significant. Full model specifications and sensitivity analyses are provided in Supplementary material 14.

For self-reported SPiN performance, no significant interactions between SES and any of the FFR contrasts were detected; therefore, additive specifications were retained for interpretation. SES effects were uniformly non-significant across all SES-only models. When the FFR predictors were added to the models, SES remained non-significant in all additive specifications, and none of the FFR contrasts accounted for meaningful variance in self-reported SPiN performance. The onset-latency contrast showed a non-significant trend (*β* = 0.377, *p* = 0.109), while the transition-latency contrast (*β* = −0.037, *p* = 0.670), the transition stimulus-to-response correlation contrast (*β* = 4.747, *p* = 0.118), the steady-state correlation contrast (*β* = −0.462, *p* = 0.896), and the full-stimulus correlation contrast (*β* = 4.949, *p* = 0.156) were all non-significant. Full model specifications and sensitivity analyses are provided in Supplementary material 15.

## 4 Discussion

### 4.1 Differential susceptibility to noise-related degradation

The primary aim of the present study was to determine whether SES is associated with differential susceptibility to noise-related degradation in neural speech encoding. A significant interaction between SES and noise condition was found for onset and transition latencies, as well as for stimulus-to-response correlations in the transition, steady-state, and full-stimulus segments. This interaction suggests that while noise adversely affected both groups, individuals in the high-SES group were significantly less impacted. Previous studies by Skoe et al. (2013) and Anderson et al. (2013b) had already reported an association between SES and the quality of FFRs under both quiet and noisy conditions. However, to our knowledge, no previous study has examined whether SES modulates the degree of vulnerability to noise interference in speech encoding.

The finding that SES modulates noise-related delays in both onset and transition latencies indicates that SES influences multiple stages of neural timing. These two components reflect distinct physiological processes: onset latencies capture the initial neural synchrony to sound onset, whereas transition latencies index the encoding of rapid formant changes (Anderson and Kraus, 2010; Anderson et al., 2010). Functionally, these temporal windows jointly support speech processing, with onset timing facilitating rapid alignment to the acoustic stream and transition timing enabling the tracking of spectrotemporal cues crucial for phonetic identification (Skoe and Kraus, 2010). Neurophysiologically, both onset and transition responses depend on subcortical phase-locked activity, and their rapid timing features are consistent with generation in the inferior colliculus, identified as the dominant source of the human FFR and necessary for its transient components (Coffey et al., 2019). Accordingly, SES-related differences in onset and transition timing may reflect subtle variations in subcortical processing, particularly within the inferior colliculus, and these timing advantages may allow higher-SES individuals to maintain more stable neural representations of speech under noise.

Although onset and transition responses originate from similar subcortical mechanisms, they differ substantially in their susceptibility to external and internal influences. Under noisy conditions, transition latencies exhibit greater delays than onset peaks, consistent with the heightened vulnerability of mid-syllabic, rapidly changing cues to energetic masking (Chandrasekaran et al., 2009; Parbery-Clark et al., 2009a; Anderson et al., 2013c). Even in quiet, transition timing remains more sensitive to listener-related factors, including age-related declines in temporal precision (Anderson et al., 2012) and enhancements associated with musical experience (Wong et al., 2007). These findings suggest that the transition region is doubly vulnerable, being more affected by adverse acoustic environments and more malleable to experiential and maturational influences across the life span. In the present study, individuals from higher-SES backgrounds exhibited reduced susceptibility at both encoding stages, preserving onset robustness and mitigating delays in the more fragile transition period. This pattern supports the view that transition timing, due to its experiential sensitivity, may also be shaped by socioeconomic context.

In addition to latency effects, an interaction between SES and noise was also observed for stimulus-to-response correlations, which reflect the overall fidelity of the neural representation of speech across the auditory pathway (Anderson and Kraus, 2010; Anderson et al., 2010). From a functional perspective, higher stimulus-to-response correlations indicate a more faithful transcription of the acoustic signal, whereas lower values reflect diminished stability in how the auditory system tracks the temporal structure of speech. Neurophysiologically, these correlations are shaped largely by phase-locked activity from subcortical sources such as the inferior colliculus. However, evidence from magnetoencephalography and electroencephalography points to cortical FFR generators at F_0_ and envelope frequencies, suggesting that this measure may also reflect contributions from higher auditory levels and capture, at least in part, integrated speech encoding across multiple stages of the system (Coffey et al., 2016, 2019; Gorina-Careta et al., 2021). The greater noise-related reduction in correlation strength among low-SES participants suggests that SES disparities extend beyond neural timing to broader declines in response fidelity. This may reflect subtle differences in phase-locked mechanisms across subcortical and cortical levels, resulting in less stable neural representations of speech in noise.

This pattern of SES-related differences in susceptibility to noise-related degradation aligns with prior evidence showing that certain life experiences can confer greater resilience in neural speech encoding under adverse conditions. Although Parbery-Clark et al. (2009a) did not examine SES, their findings illustrate a comparable interaction effect. In their study, musicians who began training before the age of seven and accumulated at least 10 years of practice showed less disruption in FFRs than non-musicians. This advantage was observed when participants were tested both in quiet and in six-talker babble at +10 dB SNR. While noise affected both groups, musicians demonstrated smaller delays in onset and transition latencies, as well as slightly better stimulus-to-response correlations in the steady-state portion. No group differences were observed for amplitudes, F_0_ encoding, harmonic strength, or quiet-to-noise correlations.

A similar pattern of differential susceptibility to signal degradation was reported by Bidelman and Krishnan (2010), who compared musicians with at least 8 years of continuous musical training beginning before age ten to age-matched non-musicians in their responses to reverberation. All participants were between 20 and 30 years old. Using a synthetic /i/ vowel, FFRs were recorded under four conditions: no reverberation (dry) and three increasing reverberation levels (0.7 s, 0.8 s, and 0.9 s). As in studies using noise as the degrading factor, musicians showed stronger responses in the dry condition and maintained higher F_0_ and lower-harmonic encoding magnitudes across all levels of reverberation. Notably, a significant interaction between group and condition emerged in the dry-to-reverberant correlations: while both groups exhibited some degradation, musicians preserved waveform morphology even under severe reverberation, whereas non-musicians showed a progressive decline in similarity to the baseline. Although reverberation and noise differ in their acoustic properties, the findings of Parbery-Clark et al. (2009a) and Bidelman and Krishnan (2010) together show that adverse listening conditions reduce the precision of temporal and spectral encoding, and that stronger baseline neural representations, in this case attributed to musical training, may reduce susceptibility to such degradation.

However, evidence in the literature is not entirely consistent, and some studies have failed to observe such experiential advantages. Whiteford et al. (2025) reported no association between musical training and FFR encoding measures under multi-talker babble. Taken together, these findings raise the possibility that resilience effects may not be attributable solely to discrete or domain-specific experiential factors, but could depend on broader and more cumulative aspects of life context. From this perspective, SES differs fundamentally from discrete forms of training such as music. Rather than indexing a specific structured activity, SES reflects long-term exposure to differences in cognitive and linguistic stimulation, chronic stress, and disparities in health-related conditions that influence neural development (Farah, 2017). Such cumulative influences may operate at a broader ecological level than discrete experiential categories, offering a potential explanation for why SES-related differences in neural speech encoding are observed even when other experiential effects vary across studies.

Participants in the present study not only exhibited differential noise-related degradation of FFR responses but also showed baseline differences under quiet conditions. These results are consistent with those of Skoe et al. (2013), who compared adolescents aged 14–15 years from low-SES backgrounds (mothers with a high school diploma or less) and high-SES backgrounds (mothers with postsecondary education). These authors reported that the high-SES group showed greater amplitude in the encoding of the first formant (264–656 Hz) and higher stimulus-to-stimulus response consistency under quiet conditions. However, unlike the present study, they also observed group differences in the amplitude of the pre-stimulus interval, with greater spontaneous neural activity in the low-SES group. The authors attributed these findings to auditory impoverishment associated with SES disadvantage, characterized by reduced exposure to linguistically rich environments and increased exposure to unstructured ambient noise, leading to noisier, less stable, and less efficient auditory responses.

Overall, the present findings support the idea that individuals from different SES backgrounds, operationalized through maternal education, undergo distinct long-term auditory experiences that shape cortical and subcortical function as reflected in the FFR. In favorable contexts, these experiences resemble the functional adaptations observed in musicians (Musacchia et al., 2007; Parbery-Clark et al., 2009b; Bidelman and Krishnan, 2010). Similar to musical training, individuals from higher SES backgrounds may benefit from a form of prolonged auditory enrichment during early life, characterized by richer linguistic stimulation (Cartmill et al., 2013; Fernald et al., 2013; Weisleder and Fernald, 2013) and reduced exposure to environmental noise (Carrier et al., 2016; Casey et al., 2017; Rahman et al., 2022). This enriched auditory experience may help explain the SES-related differences observed in neural encoding by shaping the stability and precision of auditory processing in ways that endure into adolescence and adulthood.

Conversely, unfavorable auditory experiences can exacerbate disparities in neural encoding. Evidence from severe forms of auditory deprivation demonstrates the importance of early input quality. Cochlear implant outcomes are substantially poorer when implantation occurs after the window of maximal plasticity in early childhood, indicating a reduced capacity to establish stable auditory representations once this period has passed (Sharma and Campbell, 2011; Bidelman and Momtaz, 2021). The same developmental principle is evident in more common and transient forms of deprivation. Children with recurrent otitis media, despite eventually recovering peripheral hearing, show long-lasting alterations in brainstem encoding, including delayed FFR latencies and reduced amplitudes (Colella-Santos et al., 2019; Borges et al., 2020). These findings indicate that even relatively mild disruptions to the auditory signal during sensitive periods can have enduring neural consequences. Low-SES populations are not only more likely to encounter environmental forms of auditory deprivation, such as reduced linguistic stimulation and elevated ambient noise, but also exhibit higher rates of early-life otitis media (Pershad et al., 2024). Together, these factors offer a plausible pathway through which SES disadvantage can shape long-term neural encoding.

Building on the idea that early auditory experiences can produce long-lasting effects on neural encoding, the SES-related differences observed in the present study indicate that such influences endure even when individuals later converge in educational attainment. Although all participants were enrolled in higher education programs, clear SES differences in neural encoding remained. This indicates that tertiary education does not fully compensate for disparities established during critical periods of auditory development. Supporting this view, prior evidence shows that the long-term effects of early auditory enrichment, such as musical training, remain evident decades after formal practice has ceased. For example, White-Schwoch et al. (2013) demonstrated that older adults with more years of childhood musical training continued to display enhanced neural timing and amplitudes, even in noise, compared with peers with little or no training. Together, these findings suggest that early socioeconomic and experiential factors shape neural speech encoding in ways that persist into adulthood and are not readily reversed by later improvements in socioeconomic circumstances, including educational attainment.

### 4.2 Behavioral and self-reported SPiN

The secondary aim was to examine whether SES-linked differential effects of noise on neural responses are mirrored by corresponding SES-related differences in behavioral and self-reported SPiN performance. Overall, the results did not indicate clear SES-related differences in either behavioral or self-reported SPiN performance. Beyond SES, two FFR parameters showed robust associations with behavioral SPiN performance. The onset-latency contrast and the full-stimulus stimulus-to-response correlation contrast were significant predictors, with longer onset delays and reduced neural fidelity corresponding to higher SRT, that is, poorer performance. Although SES did not independently predict behavioral SPiN performance, SES was associated with differences in FFR measures that themselves significantly predicted this behavioral outcome, indicating a potential indirect link between SES and listening performance via neural encoding mechanisms.

These findings align with previous evidence indicating that neural timing and response fidelity under noisy conditions are reliable predictors of SPiN performance. Delays in onset timing, reduced precision in formant-transition encoding, and weaker stimulus-to-response correlations have consistently been linked to poorer outcomes on sentence-based tasks such as HINT and QuickSIN (Anderson and Kraus, 2010; Anderson et al., 2010; Parbery-Clark et al., 2009a; Parbery-Clark et al., 2009b; Song et al., 2011; Thompson et al., 2019; Skoe and Kraus, 2024), although, these tasks engage both sensory and higher-order linguistic processes. In contrast, the monosyllabic materials used in the present study impose minimal cognitive demands, offering a more direct index of sensory encoding. This methodological distinction may help explain the strong associations observed between FFR markers, particularly onset-latency delays and reduced full-stimulus stimulus-to-response correlations, and behavioral SPiN performance, reinforcing the functional relevance of noise-related variability in neural encoding.

Regarding self-reported SPiN performance, neither SES nor the FFR contrast showed a significant effect. The relatively young age of the participants may partly explain the absence of detectable differences. While a portion of individuals with clinically normal hearing report SPiN challenges (Tremblay et al., 2015; Smith et al., 2019), such difficulties tend to become more pronounced with advancing age (Weissgerber et al., 2022). This is consistent with prior findings by Anderson et al. (2013a) who reported that FFR measures significantly predicted self-reported SPiN performance on the SSQ in a sample of adults aged 45 to 78 years. However, the younger adult sample in the present study, aged 18 to 30 years, did not yield any neural predictors of perceived difficulty, suggesting that age-related increases in everyday communication challenges may be necessary for such associations to emerge.

In addition to age, differences in the version and administration of the self-report instrument may have contributed to the discrepancy in findings. Anderson et al. (2013a) used the original SSQ, which includes 49 items covering a broad range of everyday listening situations (Noble et al., 2013), and focused their analysis on the 14-item Speech subscale. In contrast, the present study employed the abbreviated SSQ-12, which contains only 12 items in total and was designed to function as a unitary global measure of perceived hearing difficulties (Cañete et al., 2022). Although this version also includes a subset of four items related to speech perception, independent analysis of these items is not recommended due to their limited number and reduced coverage of communicative contexts. These methodological differences in the present study may have reduced the sensitivity of the self-report measure to detect subtle SES-related differences in perceived listening difficulty.

### 4.3 Limitations and projections

One limitation of the present study was the use of maternal education as the sole indicator of SES. While this measure is widely validated and has proven to be a strong predictor of early linguistic and cognitive development, it does not capture the full multidimensional nature of socioeconomic disadvantage, which includes factors such as household income, parental occupation, and neighborhood characteristics. Although maternal education reflects critical aspects of the early environment, it does not allow for the disentanglement of specific SES components that may contribute to the observed neurophysiological differences. As noted by Skoe et al. (2013) maternal education is a particularly sensitive proxy for early auditory and linguistic input quality; nonetheless, it should be understood as a partial approximation that cannot fully account for the complex SES conditions shaping auditory development. Future research should incorporate a more comprehensive SES assessments to further understand how distinct socioeconomic dimensions shape auditory system development.

A second limitation concerns the sample, which was restricted to young adults currently enrolled in higher education programs. This constitutes a relatively homogeneous group that is not representative of the broader Chilean population. Such restriction likely reduced variability in cognitive stimulation and intellectual engagement, factors known to modulate the relationship between SES and SPiN performance (Anderson et al., 2013b). As a result, SES-related differences in behavioral measures may have been attenuated, even if neurophysiological indices continued to reveal clear group effects. Future studies should include more socioeconomically diverse samples to better capture the full range of SES-related variability in both neural and behavioral outcomes.

## 5 Conclusion

The results of the present study evidence that individuals from higher-SES backgrounds exhibit greater neural resilience to background noise, as reflected in smaller delays in onset and transition latencies and more stable stimulus-to-response correlations. Beyond this interaction effect, consistent SES-related differences were also observed under quiet conditions, with the low-SES group showing delayed transition latencies, lower broadband amplitudes, reduced harmonic encoding, and weaker stimulus-to-response correlations. These neural disparities were not mirrored by SES-related differences in either behavioral or self-reported SPiN performance. However, specific FFR measures, particularly onset latency and stimulus-to-response correlations, emerged as significant predictors of behavioral outcomes, reinforcing the functional relevance of noise-related variability in neural encoding. Overall, the findings highlight SES as a meaningful determinant of auditory brainstem function and point to enduring influences of early environmental experience on the precision of neural speech encoding across listening conditions.

## Data Availability

The raw data supporting the conclusions of this article will be made available by the authors, without undue reservation.

## Footnotes

1
https://brainvolts.northwestern.edu
