# Supplementary material for: Neural speech encoding advantages associated with higher socioeconomic status extend to noise conditions with differential susceptibility
Source: Front Psychol. 2026 Mar 9;17:1760305. doi: 10.3389/fpsyg.2026.1760305 (PMC13006642; doi:10.3389/fpsyg.2026.1760305)
Supplement: Supplementary file 1 [file Supplementary_file_1.docx]

Supplementary Material

# Supplementary Material 1. Correlation matrices between electrophysiological measures of the FFR, behavioral SPiN performance, self-perceived SPiN, and cognitives variables.

## Table S1.1. Correlation matrix between FFR parameters in the silence condition, behavioral SPiN, self-perceived SPiN, and cognitive variables.

| Variable | Working Memory | Vocabulary | P9 Latency | Transition Latencies | Steady-State Latencies | Pre-stimulus RMS | Transition RMS | Steady-State RMS | Full-Stimulus-RMS | F0 | H2 | H3 | Cor Stim-Resp Transition | Cor Stim-Resp Steady-State | Cor Stim-Resp Full-Stimulus | Cor Resp-Resp Transition | Cor Resp-Resp Steady-State | Cor Resp-Resp Full-Stimulus | Speech Recognition Threshold | SSQ-12 |
| --- | --- | --- | --- | --- | --- | --- | --- | --- | --- | --- | --- | --- | --- | --- | --- | --- | --- | --- | --- | --- |
| Working Memory | 1.000 |  |  |  |  |  |  |  |  |  |  |  |  |  |  |  |  |  |  |  |
| Vocabulary | **0.547***** | 1.000 |  |  |  |  |  |  |  |  |  |  |  |  |  |  |  |  |  |  |
| p9 Latency | 0.129 | -0.045 | 1.000 |  |  |  |  |  |  |  |  |  |  |  |  |  |  |  |  |  |
| Transition Latencies | -0.193 | -0.047 | **0.244*** | 1.000 |  |  |  |  |  |  |  |  |  |  |  |  |  |  |  |  |
| Steady-State-Latencies | -0.069 | -0.154 | **0.333**** | **0.500***** | 1.000 |  |  |  |  |  |  |  |  |  |  |  |  |  |  |  |
| Pre-stimulus RMS | 0.180 | 0.055 | 0.013 | **-0.127** | 0.008 | 1.000 |  |  |  |  |  |  |  |  |  |  |  |  |  |  |
| Transition RMS | **0.519***** | 0.224 | -0.078 | **-0.459***** | -0.098 | 0**.322**** | 1.000 |  |  |  |  |  |  |  |  |  |  |  |  |  |
| Steady-State RMS | **0.527***** | 0.205 | -0.064 | **-0.443**** | -0.093 | **0.300*** | **0.982***** | 1.000 |  |  |  |  |  |  |  |  |  |  |  |  |
| Full-Stimulus-RMS | **0.522***** | 0.213 | -0.067 | **-0.443**** | -0.092 | **0.314**** | **0.988***** | **0.999***** | 1.000 |  |  |  |  |  |  |  |  |  |  |  |
| F0 | **0.336**** | 0.100 | -0.184 | -0.020 | -0.104 | 0.169 | **0.942***** | **0.963***** | **0.959***** | 1.000 |  |  |  |  |  |  |  |  |  |  |
| H2 | **0.309*** | 0.201 | -0.162 | -0.050 | -0.152 | 0.192 | **0.802***** | **0.875***** | **0.874***** | **0.775***** | 1.000 |  |  |  |  |  |  |  |  |  |
| H3 | 0.179 | 0.045 | -0.179 | 0.066 | -0.099 | 0.137 | **0.791***** | **0.854***** | **0.846***** | **0.817***** | **0.741***** | 1.000 |  |  |  |  |  |  |  |  |
| Transition Stim-Resp Cor | 0.004 | 0.098 | -0.145 | -0.147 | **-0.293*** | 0.035 | 0.122 | 0.122 | 0.124 | 0.182 | **0.292*** | 0.126 | 1.000 |  |  |  |  |  |  |  |
| Steady-State Stim-Resp Cor | 0.180 | 0.152 | -0.081 | -0.206 | -0.178 | -0.029 | 0.094 | 0.115 | 0.112 | 0.096 | 0.240 | -0.035 | 0.004 | 1.000 |  |  |  |  |  |  |
| Full-Stimulus Stim-Resp Cor | **0.280*** | 0.018 | -0.064 | **-0.244*** | -0.216 | 0.029 | 0.219 | **0.264*** | **0.254*** | 0.230 | **0.334**** | 0.047 | **0.296*** | **0.704***** | 1.000 |  |  |  |  |  |
| Transition Resp-Resp Cor | 0.033 | 0.074 | -1.190 | -0.185 | **-0.284*** | -0.182 | **0.323**** | **0.289*** | **0.296*** | 0.238 | 0.225 | 0.204 | 0.116 | 0.138 | 0.136 | 1.000 |  |  |  |  |
| Steady-State Resp-Resp Cor | 0.053 | -0.094 | -0.033 | -0.069 | -0.153 | -0.192 | **0.298*** | **0.333**** | **0.326**** | **0.250*** | **0.264*** | **0.326**** | 0.097 | 0.174 | **0.242*** | **0.668***** | 1.000 |  |  |  |
| Full-Stimulus Resp-Resp Cor | 0.030 | 0.009 | -0.114 | -0.133 | -0.203 | -0.215 | **0.345**** | **0.354**** | **0.353**** | **0.280*** | **0.281*** | **0.319*** | 0.108 | 0.183 | 0.213 | **0.842***** | **0.950***** | 1.000 |  |  |
| Speech Recognition Threshold | -0.069 | -0.102 | -0.091 | 0.029 | -0.019 | 0.093 | -0.087 | -0.083 | -0.083 | -0.028 | -0.030 | -0.002 | 0.075 | 0.101 | **0.257*** | -0.084 | 0.018 | -0.121 | 1.000 |  |
| SSQ-12 | -0.124 | -0.061 | -**0.240*** | -0.049 | -0.131 | -0.043 | -0.193 | -0.180 | -0.182 | -0.095 | 0.035 | -0.112 | -0.075 | 0.024 | 0.078 | -0.189 | -0.121 | -0.135 | **0.238*** | 1.000 |

* p < 0.05; ** p < 0.01; *** p < 0.001. Transition and steady-state latencies were reduced to their first principal component scores through principal component analysis (PCA) prior to correlation analysis to reduce dimensionality. RMS: root mean square; F0: fundamental frequency; H2: second harmonic; H3: third harmonic; Cor: correlation; Stim: stimulus.

## Table S1.2. Correlation matrix between FFR parameters in the noise condition, behavioral SPiN, self-perceived SPiN, and cognitive variables.

| Variable | Working Memory | Vocabulary | P9 Latency | Transition Latencies | Steady-State Latencies | Pre-stimulus RMS | Transition RMS | Steady-State RMS | Full-Stimulus-RMS | F0 | H2 | H3 | Cor Stim-Resp Transition | Cor Stim-Resp Steady-State | Cor Stim-Resp Full-Stimulus | Cor Resp-Resp Transition | Cor Resp-Resp Steady-State | Cor Resp-Resp Full-Stimulus | Speech Recognition Threshold | SSQ-12 |
| --- | --- | --- | --- | --- | --- | --- | --- | --- | --- | --- | --- | --- | --- | --- | --- | --- | --- | --- | --- | --- |
| Working Memory | 1.000 |  |  |  |  |  |  |  |  |  |  |  |  |  |  |  |  |  |  |  |
| Vocabulary | **0.547***** | 1.000 |  |  |  |  |  |  |  |  |  |  |  |  |  |  |  |  |  |  |
| p9 Latency | -0.196 | 0.000 | 1.000 |  |  |  |  |  |  |  |  |  |  |  |  |  |  |  |  |  |
| Transition Latencies | -0.216 | -0.089 | **0.488***** | 1.000 |  |  |  |  |  |  |  |  |  |  |  |  |  |  |  |  |
| Steady-State-Latencies | -0.065 | 0.099 | **0.251*** | **0.337**** | 1.000 |  |  |  |  |  |  |  |  |  |  |  |  |  |  |  |
| Pre-stimulus RMS | -0.009 | 0.134 | -0.018 | -0.139 | 0.031 | 1.000 |  |  |  |  |  |  |  |  |  |  |  |  |  |  |
| Transition RMS | **0.382**** | 0.182 | **-0.259*** | **-0.356**** | -0.010 | **0.505***** | 1.000 |  |  |  |  |  |  |  |  |  |  |  |  |  |
| Steady-State RMS | **0.371**** | 0.186 | **-0.327**** | **-0.391**** | 0.016 | **0.334**** | **0.897***** | 1.000 |  |  |  |  |  |  |  |  |  |  |  |  |
| Full-Stimulus-RMS | **0.372**** | 0.184 | **-0.314*** | **-0.385**** | 0.012 | **0.402**** | **0.937***** | **0.994***** | 1.000 |  |  |  |  |  |  |  |  |  |  |  |
| F0 | **0.360**** | 0.186 | **-0.350**** | **-0.380**** | 0.056 | -0.036 | **0.686***** | **0.893***** | **0.851***** | 1.000 |  |  |  |  |  |  |  |  |  |  |
| H2 | **0.406**** | 0.166 | **-0.330**** | **-0.436***** | -0.034 | 0.048 | **0.727***** | **0.832***** | **0.811***** | **0.832***** | 1.000 |  |  |  |  |  |  |  |  |  |
| H3 | **0.368**** | 0.151 | **-0.296*** | **-0.450***** | -0.043 | -0.023 | **0.743***** | **0.842***** | **0.821***** | **0.886***** | **0.898***** | 1.000 |  |  |  |  |  |  |  |  |
| Transition Stim-Resp Cor | 0.149 | 0.172 | -0.192 | -0.046 | 0.068 | 0.065 | 0.099 | 0.068 | 0.072 | 0.103 | 0.027 | 0.043 | 1.000 |  |  |  |  |  |  |  |
| Steady-State Stim-Resp Cor | **0.277*** | 0.138 | **-0.420**** | **-0.309**** | -0.194 | -0.225 | 0.029 | 0.152 | 0.119 | **0.262*** | **0.308**** | 0.185 | **0.239*** | 1.000 |  |  |  |  |  |  |
| Full-Stimulus Stim-Resp Cor | 0.177 | 0.123 | **-0.375**** | **-0.238*** | -0.105 | **-0.297*** | 0.066 | **0.236*** | 0.186 | **0.415***** | **0.330**** | **0.250*** | **0.312**** | **0.760***** | 1.000 |  |  |  |  |  |
| Transition Resp-Resp Cor | 0.124 | 0.150 | **-0.309*** | -0.148 | -0.035 | **-0.339**** | 0.230 | **0.311*** | **0.279*** | **0.457***** | **0.295*** | **0.393**** | 0.248 | **0.368**** | **0.576***** | 1.000 |  |  |  |  |
| Steady-State Resp-Resp Cor | 0.103 | 0.168 | **-0.352**** | **-0.245*** | -0.025 | -0.183 | **0.238*** | **0.448***** | **0.394**** | **0.581***** | **0.421***** | **0.434***** | 0.207 | **0.522***** | **0.712***** | **0.687***** | 1.000 |  |  |  |
| Full-Stimulus Resp-Resp Cor | 0.138 | 0.170 | **-0.363**** | -0.222 | -0.034 | **-0.257*** | **0.252*** | **0.458***** | **0.403**** | **0.610***** | **0.428***** | **0.467***** | **0.199** | **0.509***** | **0.714***** | **0.806***** | **0.976***** | 1.000 |  |  |
| Speech Recognition Threshold | -0.069 | -0.102 | **0.301*** | 0.024 | -0.133 | **0.259*** | 0.050 | -0.015 | 0.003 | -0.123 | -0.062 | -0.095 | -0.164 | -0.166 | -0.118 | -0.188 | -0.115 | -0.127 | 1.000 |  |
| SSQ-12 | -0.124 | -0.061 | 0.011 | -0.061 | -0.083 | **0.275*** | -0.057 | -0.013 | -0.013 | -0.109 | -0.056 | -0.143 | -**0.316**** | 0.049 | -0.104 | -0.084 | -0.086 | -0.085 | **0.238*** | 1.000 |

* p < 0.05; ** p < 0.01; *** p < 0.001. Transition and steady-state latencies were reduced to their first principal component scores through principal component analysis (PCA) prior to correlation analysis to reduce dimensionality. RMS: root mean square; F0: fundamental frequency; H2: second harmonic; H3: third harmonic; Cor: correlation; Stim: stimulus.

# Supplementary Material 2. Details and comparison of linear mixed-effects models for ABRs.

**Table S2.1.** Linear mixed-effects models for the absolute ABRs latencies.

Two multilevel models were estimated: a baseline model with all available data and an outlier-removed sensitivity model. The original dataset contained 210 observations across 70 participants (clusters). For the outlier-removed model, 16 observations (7.6%) were identified as outliers, leaving 194 observations across 69 participants. Intraclass correlation coefficients (ICCs) indicated that a modest proportion of variance was attributable to between-participant differences, with estimates of 7.0% for the original model and 28.7% for the outlier-removed model, supporting the multilevel specification. The SES effect was not statistically significant in either model (p = 0.211 and p = 0.343, respectively). To assess the robustness of the findings, we estimated bootstrap confidence intervals with 10,000 replicates using cluster-resampling methods, which produced stable estimates for fixed effects.

| **Parameter** | **Full Model [95% CI]** | **p-value** | **Outlier-removed Model [95% CI]** | **p-value** |
| --- | --- | --- | --- | --- |
| Intercept | 1.534 [1.487, 1.581] | <.001 | 1.524 [1.495, 1.554] | <.001 |
| High-SES a | -0.040 [-0.103, 0.021] | .211 | -0.025 [-0.077, 0.027] | .343 |
| Wave III b | 2.164 [2.106, 2.219] | <.001 | 2.192 [2.160, 2.223] | <.001 |
| Wave V b | 3.737 [3.660, 3.830] | <.001 | 3.675 [3.624, 3.724] | <.001 |

ᵃ Reference SES is Low-SES. ᵇ Reference wave is wave I.

| **Component** | **Full Model** | **Outlier-removed Model** |
| --- | --- | --- |
| Cluster Variance (σ²) | 0.005 | 0.006 |
| Residual Variance (σ²) | 0.064 | 0.016 |
| ICC (%) | 7.0 | 28.7 |

**Table S2.2.** Linear mixed-effects models for the inter-peaks ABRs latencies.

Two multilevel models were estimated: a baseline model with all available data and an outlier-removed sensitivity model. The original dataset contained 210 observations across 70 participants (clusters). For the outlier-removed model, 8 observations (3.8%) were identified as outliers, leaving 202 observations across 69 participants. Intraclass correlation coefficients (ICCs) indicated that a moderate proportion of variance was attributable to between-participant differences, with estimates of 18.6% for the original model and 49.6% for the outlier-removed model, supporting the multilevel specification. The SES effect was not statistically significant in either model (p = 0.589 and p = 0.303, respectively). To assess the robustness of the findings, we estimated bootstrap confidence intervals with 10,000 replicates using cluster-resampling methods, which produced stable estimates for fixed effects.

| **Parameter** | **Full Model [95% CI]** | **p-value** | **Outlier-removed Model [95% CI]** | **p-value** |
| --- | --- | --- | --- | --- |
| Intercept | 2.178 [2.114, 2.236] | <.001 | 2.197 [2.157, 2.240] | <.001 |
| High-SES a | -0.027 [-0.115, 0.062] | .589 | 0.032 [-0.030, 0.098] | .303 |
| V-I Interval b | 1.573 [1.485, 1.690] | <.001 | 1.484 [1.436, 1.530] | <.001 |
| V-III Interval b | -0.592 [-0.716, -0.429] | <.001 | -0.686 [-0.745, -0.631] | <.001 |

ᵃ Reference SES is Low-SES. ᵇ Reference wave is wave I.

| **Component** | **Full Model Model** | **Outlier-removed Model** |
| --- | --- | --- |
| Cluster Variance (σ²) | 0.024 | 0.023 |
| Residual Variance (σ²) | 0.105 | 0.023 |
| ICC (%) | 18.6 | 49.6 |

**Table S2.3.** Linear mixed-effects models for the wavs ABRs amplitudes.

Two multilevel models were estimated: a baseline model with all available data and an outlier-removed sensitivity model. The original dataset contained 210 observations across 70 participants (clusters). For the outlier-removed model, 12 observations (5.7%) were identified as outliers, leaving 198 observations across 70 participants. Intraclass correlation coefficients (ICCs) indicated that a moderate proportion of variance was attributable to between-participant differences, with estimates of 21.8% for the original model and 28.9% for the outlier-removed model, supporting the multilevel specification. The SES effect was not statistically significant in the full model (p = 0.493) but approached significance in the outlier-removed model (p = 0.039). To assess the robustness of the findings, we estimated bootstrap confidence intervals with 10,000 replicates using cluster-resampling methods, which produced stable estimates for fixed effects.

| **Parameter** | **Full Model [95% CI]** | **p-value** | **Outlier-removed Model [95% CI]** | **p-value** |
| --- | --- | --- | --- | --- |
| Intercept | 0.404 [0.350, 0.459] | <.001 | 0.360 [0.323, 0.401] | <.001 |
| High-SES ᵃ | 0.016 [-0.031, 0.065] | .493 | 0.043 [0.002, 0.080] | .039 |
| Wave III ᵇ | -0.088 [-0.137, -0.041] | <.001 | -0.058 [-0.097, -0.020] | .002 |
| Wave V ᵇ | 0.125 [0.061, 0.181] | <.001 | 0.185 [0.145, 0.223] | <.001 |

ᵃ Reference SES is Low-SES. ᵇ Reference wave is wave I.

| **Component** | **Full Model** | **Outlier-removed Model** |
| --- | --- | --- |
| Cluster Variance (σ²) | 0.008 | 0.006 |
| Residual Variance (σ²) | 0.027 | 0.014 |
| ICC (%) | 21.8 | 28.9 |

**Supplementary Material 3.** Observed latency values by SES group and listening condition for different segments of the FFR elicited by a 170 ms /da/ syllable.

|  | **Low-SES** | |  | **High-SES** | |
| --- | --- | --- | --- | --- | --- |
|  | **Silence** | **Babble** |  | **Silence** | **Babble** |
| Onset |  |  |  |  |  |
| p09 | 7.96 (7.74; 8.44) | 9.22 (9.04; 9.57) |  | 7.97 (7.70; 8.16) | 8.80 (8.51; 9.27) |
|  |  |  |  |  |  |
| Transition |  |  |  |  |  |
| p23 | 22.54 (22.03; 23.13) | 23.44 (23.10; 23.76) |  | 22.42 (22.13; 23.14) | 23.10 (22.63; 23.44) |
| p32 | 32.20 (31.67; 32.95) | 33.45 (33.08; 33.72) |  | 32.03 (31.56; 32.53) | 33.15 (32.59; 33.51) |
| p42 | 42.15 (41.72; 42.47) | 42.90 (42.52; 43.64) |  | 42.03 (41.42; 42.20) | 42.64 (42.25; 43.06) |
| p52 | 51.94 (51.67; 52.05) | 53.33 (52.09; 54.00) |  | 51.81 (51.53; 51.96) | 52.61 (52.05; 53.46) |
|  |  |  |  |  |  |
| Steady-State |  |  |  |  |  |
| p62 | 62.06 (61.84; 62.29) | 62.48 (62.25; 62.80) |  | 61.95 (61.83; 62.25) | 62.33 (62.16; 62.62) |
| p72 | 72.15 (72.00; 72.25) | 72.30 (71.99; 72.68) |  | 72.07 (71.87; 72.32) | 72.34 (71.97; 72.58) |
| p82 | 82.16 (82.03; 82.27) | 82.46 (82.09; 82.78) |  | 82.12 (81.96; 82.29) | 82.38 (82.05; 82.59) |
| p92 | 92.10 (91.97; 92.29) | 92.55 (92.12; 92.91) |  | 92.06 (91.95; 92.27) | 92.48 (92.17; 92.83) |
| p102 | 102.16 (102.02; 102.32) | 102.53 (102.21; 102.70) |  | 102.15 (101.96; 102.26) | 102.49 (102.22; 102.66) |
| p112 | 112.12 (111.88; 112.30) | 112.42 (112.16; 112.74) |  | 112.09 (111.96; 112.20) | 112.32 (112.00; 112.65) |
| p122 | 122.13 (122.03; 122.28) | 122.40 (122.18; 122.74) |  | 122.14 (121.97; 122.29) | 122.44 (122.18; 122.66) |
| p132 | 132.18 (132.00; 132.23) | 132.49 (132.15; 133.07) |  | 132.07 (131.96; 132.30) | 132.45 (132.13; 132.68) |
| p142 | 142.16 (141.97; 142.26) | 142.50 (142.24; 142.68) |  | 142.12 (141.95; 142.32) | 142.43 (142.10; 142.72) |
| p152 | 152.10 (151.97; 152.25) | 152.40 (152.06; 152.97) |  | 152.14 (151.93; 152.28) | 152.51 (152.23; 152.72) |
| p162 | 162.18 (162.06; 162.30) | 162.45 (162.19; 162.83) |  | 162.15 (162.00; 162.32) | 162.49 (162.06; 162.64) |

Values are presented as the median latency in milliseconds (ms), with the 25th and 75th percentiles shown in parentheses.

**Supplementary Material 4.** Details and comparison of latency linear mixed-effects models.

**Table S4.1.** Linear mixed-effects models for the onset segment latencies.

Three multilevel models were estimated: a non-interaction model, an interaction model including the SES–condition term, and an outlier-removed sensitivity model. The original dataset contained 135 observations across 70 clusters. For the outlier-removed model, 2 observations (1.5%) were identified as outliers, leaving 133 observations across the same 70 clusters. Intraclass correlation coefficients (ICCs) indicated that a substantial proportion of variance was attributable to between-cluster differences, with estimates of 22.1% for the non-interaction model, 26.1% for the interaction model, and 24.1% for the outlier-removed model, supporting the multilevel specification. The interaction between SES and condition was statistically significant in the interaction model, which was therefore retained for interpretation. To assess the robustness of the findings, we estimated an outlier-removed counterpart as a sensitivity model, which produced highly consistent results.

| **Parameter** | **Non-interaction Model [95% CI]** | **p-value** | **Interaction Model [95% CI]** | **p-value** | **Outlier-removed Model [95% CI]** | **p-value** |
| --- | --- | --- | --- | --- | --- | --- |
| Intercept | 8.200 [8.069, 8.332] | <.001 | 8.104 [7.963, 8.244] | <.001 | 8.104 [7.967, 8.242] | <.001 |
| High-SES ^a^ | -0.335 [-0.484, -0.184] | <.001 | -0.143 [-0.329, 0.046] | .143 | -0.178 [-0.348, 0.013] | .070 |
| Babble ^b^ | 1.067 [0.896, 1.254] | <.001 | 1.267 [1.076, 1.479] | <.001 | 1.221 [1.044, 1.412] | <.001 |
| High-SES ^a^ × Babble ^b^ | - | - | -0.402 [-0.726, -0.108] | .008 | -0.322 [-0.641, -0.036] | .027 |

ᵃ Reference SES is Low-SES. ᵇ Reference condition is silence.

| **Component** | **Non-interaction Model** | **Interaction Model** | **Outlier-removed Model** |
| --- | --- | --- | --- |
| Cluster Variance (σ²) | 0.064 | 0.073 | 0.061 |
| Residual Variance (σ²) | 0.226 | 0.208 | 0.194 |
| ICC (%) | 22.1 | 26.1 | 24.1 |

**Table S4.2.** Linear mixed-effects models for the transition segment latencies.

Three multilevel models were estimated: a non-interaction model, an interaction model including the SES–condition term, and an outlier-removed sensitivity model. The original dataset contained 560 observations across 70 clusters. For the outlier-removed model, 12 observations (2.1%) were identified as outliers, leaving 548 observations across the same 70 clusters. Intraclass correlation coefficients (ICCs) indicated that a substantial proportion of variance was attributable to between-cluster differences, with estimates of 24.4% for the non-interaction model, 24.6% for the interaction model, and 22.8% for the outlier-removed model, supporting the multilevel specification. The interaction between SES and condition was statistically significant in the interaction model, which was therefore retained for interpretation. To assess the robustness of the findings, we estimated an outlier-removed counterpart as a sensitivity model, which produced highly consistent results.

| **Parameter** | **Non-interaction Model [95% CI]** | **p-value** | **Interaction Model [95% CI]** | **p-value** | **Outlier-removed Model [95% CI]** | **p-value** |
| --- | --- | --- | --- | --- | --- | --- |
| Intercept | -0.407 [-0.646, -0.159] | .002 | -0.479 [-0.719, -0.235] | <.001 | -0.518 [-0.751, -0.275] | <.001 |
| High-SES ^a^ | -0.331 [-0.500, -0.158] | <.001 | -0.188 [-0.350, -0.028] | .023 | -0.171 [-0.333, -0.019] | .030 |
| Babble ^b^ | 0.954 [0.845, 1.073] | <.001 | 1.098 [0.934, 1.281] | <.001 | 1.054 [0.902, 1.221] | <.001 |
| Peak | 1.012 [1.006, 1.017] | <.001 | 1.012 [1.006, 1.017] | <.001 | 1.013 [1.007, 1.018] | <.001 |
| High-SES ^a^ × Babble ^b^ | - | - | -0.287 [-0.510, -0.075] | .009 | -0.235 [-0.453, -0.024] | .032 |

ᵃ Reference SES is Low-SES. ᵇ Reference condition is silence.

| **Component** | **Non-interaction Model** | **Interaction Model** | **Outlier-removed Model** |
| --- | --- | --- | --- |
| Cluster Variance (σ²) | 0.160 | 0.161 | 0.136 |
| Residual Variance (σ²) | 0.497 | 0.492 | 0.459 |
| ICC (%) | 24.4 | 24.6 | 22.8 |

**Table S4.3.** Linear mixed-effects models for the steady-state segment latencies.

Three multilevel models were estimated: a non-interaction model, an interaction model including the SES–condition term, and an outlier-removed sensitivity model. The original dataset contained 1,540 observations across 70 clusters. For the outlier-removed model, 160 observations (10.4%) were identified as outliers, leaving 1,380 observations across the same 70 clusters. Intraclass correlation coefficients (ICCs) indicated that a substantial proportion of variance was attributable to between-cluster differences, with estimates of 36.9% for the non-interaction model, 36.9% for the interaction model, and 38.8% for the outlier-removed model, supporting the multilevel specification. The interaction between SES and condition was not statistically significant in the interaction model, which was therefore retained for interpretation. To assess the robustness of the findings, we estimated an outlier-removed counterpart as a sensitivity model, which produced highly consistent results.

| **Parameter** | **Non-interaction Model [95% CI]** | **p-value** | **Interaction Model [95% CI]** | **p-value** | **Outlier-removed Model [95% CI]** | **p-value** |
| --- | --- | --- | --- | --- | --- | --- |
| Intercept | 0.129 [-0.003, 0.263] | .056 | 0.119 [-0.011, 0.247] | .072 | 0.040 [-0.057, 0.140] | .407 |
| High-SES ^a^ | -0.089 [-0.237, 0.058] | .235 | -0.068 [-0.204, 0.070] | .303 | -0.016 [-0.107, 0.075] | .722 |
| Babble ^b^ | 0.368 [0.269, 0.491] | <.001 | 0.389 [0.239, 0.601] | <.001 | 0.291 [0.240, 0.343] | <.001 |
| Peak | 1.001 [1.000, 1.001] | <.001 | 1.001 [1.000, 1.001] | <.001 | 1.001 [1.000, 1.001] | <.001 |
| High-SES ^a^ × Babble ^b^ | - | - | -0.041 [-0.284, 0.162] | .748 | - | - |

ᵃ Reference SES is Low-SES. ᵇ Reference condition is silence.

| **Component** | **Non-interaction Model** | **Interaction Model** | **Outlier-removed Model** |
| --- | --- | --- | --- |
| Cluster Variance (σ²) | 0.151 | 0.151 | 0.057 |
| Residual Variance (σ²) | 0.259 | 0.259 | 0.090 |
| ICC (%) | 36.9 | 36.9 | 38.8 |

**Supplementary Material 5.** Observed Root Mean Square (RMS) values by SES group and listening condition for different FFR segments elicited by a 170 ms /da/ syllable.

|  | **Low-SES** | |  | **High-SES** | |
| --- | --- | --- | --- | --- | --- |
|  | **Silence** | **Babble** |  | **Silence** | **Babble** |
| Pre-stimulus | 0.117 (0.095; 0.157) | 0.114 (0.092; 0.153) |  | 0.133 (0.103; 0.159) | 0.134 (0.111; 0.165) |
| Transition | 0.214 (0.172; 0.229) | 0.143 (0.119; 0.165) |  | 0.228 (0.194; 0.268) | 0.169 (0.135; 0.199) |
| Steady-State | 0.197 (0.159; 0.227) | 0.164 (0.131; 0.201) |  | 0.233 (0.201; 0.289) | 0.191 (0.170; 0.236) |
| Full-stimulus | 0.203 (0.163; 0.221) | 0.159 (0.127; 0.181) |  | 0.220 (0.197; 0.283) | 0.183 (0.160; 0.228) |

Values are presented as the median RMS in microvolts (µV), with the 25th and 75th percentiles shown in parentheses.

**Supplementary Material 6.** Details and comparisons of linear mixed-effects models for RMS broadband magnitude.

**Table S6.1.** Linear mixed-effects model for pre-stimulus RMS broadband magnitude.

Three multilevel models were estimated: a non-interaction model, an interaction model including the SES–condition term, and an outlier-removed sensitivity model. The original dataset contained 140 observations across 70 clusters. For the outlier-removed model, 3 observations (2.1%) were identified as outliers, leaving 137 observations across the same 70 clusters. Intraclass correlation coefficients (ICCs) indicated that a substantial proportion of variance was attributable to between-cluster differences, with estimates of 32.0% for the non-interaction model, 31.6% for the interaction model, and 38.2% for the outlier-removed model, supporting the multilevel specification. The interaction between SES and condition was not statistically significant in the interaction model, which was therefore retained for interpretation. To assess the robustness of the findings, we estimated an outlier-removed counterpart as a sensitivity model, which produced highly consistent results.

| **Parameter** | **Non-interaction Model [95% CI]** | **p-value** | **Interaction Model [95% CI]** | **p-value** | **Outlier-removed Model [95% CI]** | **p-value** |
| --- | --- | --- | --- | --- | --- | --- |
| Intercept | -2.175 [-2.272, -2.068] | <.001 | -2.158 [-2.271, -2.035] | <.001 | -2.175 [-2.274, -2.069] | <.001 |
| High-SES ^a^ | 0.136 [0.021, 0.240] | .022 | 0.101 [-0.041, 0.242] | .170 | 0.135 [0.029, 0.238] | .012 |
| Babble ^b^ | 0.051 [-0.049, 0.153] | .327 | 0.016 [-0.142, 0.178] | .861 | 0.010 [-0.082, 0.100] | .832 |
| High-SES ^a^ × Babble ^b^ | - | - | 0.070 [-0.136, 0.274] | .497 | - | - |

ᵃ Reference SES is Low-SES. ᵇ Reference condition is silence.

| **Component** | **Non-interaction Model** | **Interaction Model** | **Outlier-removed Model** |
| --- | --- | --- | --- |
| Cluster Variance (σ²) | 0.044 | 0.044 | 0.046 |
| Residual Variance (σ²) | 0.094 | 0.095 | 0.074 |
| ICC (%) | 32.0 | 31.6 | 38.2 |

**Table S6.2.** Linear mixed-effects model for transition RMS broadband magnitude.

Three multilevel models were estimated: a non-interaction model, an interaction model including the SES–condition term, and an outlier-removed sensitivity model. The original dataset contained 140 observations across 70 clusters. For the outlier-removed model, 12 observations (8.6%) were identified as outliers, leaving 128 observations across 66 clusters. Intraclass correlation coefficients (ICCs) indicated that a substantial proportion of variance was attributable to between-cluster differences, with estimates of 51.3% for the non-interaction model, 51.3% for the interaction model, and 26.8% for the outlier-removed model, supporting the multilevel specification. The interaction between SES and condition was not statistically significant in the interaction model, which was therefore retained for interpretation. To assess the robustness of the findings, we estimated an outlier-removed counterpart as a sensitivity model, which produced highly consistent results.

| **Parameter** | **Non-interaction Model [95% CI]** | **p-value** | **Interaction Model [95% CI]** | **p-value** | **Outlier-removed Model [95% CI]** | **p-value** |
| --- | --- | --- | --- | --- | --- | --- |
| Intercept | -2.246 [-2.706, -1.471] | <.001 | -2.270 [-2.746, -1.482] | <.001 | -1.365 [-1.579, -1.135] | <.001 |
| High-SES ᵃ | 0.159 [0.048, 0.260] | .006 | 0.207 [0.066, 0.347] | .006 | 0.134 [0.070, 0.205] | <.001 |
| Babble ᵇ | -0.341 [-0.427, -0.249] | <.001 | -0.293 [-0.416, -0.152] | <.001 | -0.319 [-0.389, -0.258] | <.001 |
| Working Memory | 0.027 [-0.005, 0.045] | .081 | 0.027 [-0.005, 0.045] | .081 | -0.011 [-0.020, -0.003] | .007 |
| High-SES ^a^ × Babble ^b^ |  |  | -0.095 [-0.282, 0.075] | .289 |  |  |

ᵃ Reference SES is Low-SES. ᵇ Reference condition is silence.

| **Component** | **Non-interaction Model** | **Interaction Model** | **Outlier-removed Model** |
| --- | --- | --- | --- |
| Cluster Variance (σ²) | 0.079 | 0.079 | 0.013 |
| Residual Variance (σ²) | 0.075 | 0.075 | 0.037 |
| ICC (%) | 51.3 | 51.3 | 26.8 |

**Table S6.3.** Linear mixed-effects model for steady-state RMS broadband magnitude.

Three multilevel models were estimated: a non-interaction model, an interaction model including the SES–condition term, and an outlier-removed sensitivity model. The original dataset contained 140 observations across 70 clusters. For the outlier-removed model, 11 observations (7.9%) were identified as outliers, leaving 129 observations across 67 clusters. Intraclass correlation coefficients (ICCs) indicated that a substantial proportion of variance was attributable to between-cluster differences, with estimates of 61.0% for the non-interaction model, 61.3% for the interaction model, and 47.9% for the outlier-removed model, supporting the multilevel specification. The interaction between SES and condition was not statistically significant in the interaction model (p = .173), which was therefore retained for interpretation. To assess the robustness of the findings, we estimated an outlier-removed counterpart as a sensitivity model, which produced highly consistent results.

| **Parameter** | **Non-interaction Model [95% CI]** | **p-value** | **Interaction Model [95% CI]** | **p-value** | **Outlier-removed Model [95% CI]** | **p-value** |
| --- | --- | --- | --- | --- | --- | --- |
| Intercept | -2.339 [-2.857, -1.485] | <.001 | -2.369 [-2.892, -1.497] | <.001 | -1.520 [-1.764, -1.284] | <.001 |
| High-SES ᵃ | 0.207 [0.084, 0.318] | .002 | 0.266 [0.119, 0.409] | <.001 | 0.184 [0.107, 0.268] | <.001 |
| Babble ᵇ | -0.205 [-0.291, -0.117] | <.001 | -0.146 [-0.257, -0.014] | .032 | -0.190 [-0.254, -0.132] | <.001 |
| Working Memory | 0.030 [-0.005, 0.051] | .082 | 0.030 [-0.005, 0.051] | .082 | -0.006 [-0.015, 0.004] | .256 |
| High-SES ^a^ × Babble ^b^ |  |  | -0.118 [-0.299, 0.049] | .173 |  |  |

ᵃ Reference SES is Low-SES. ᵇ Reference condition is silence.

| **Component** | **Non-interaction Model** | **Interaction Model** | **Outlier-removed Model** |
| --- | --- | --- | --- |
| Cluster Variance (σ²) | 0.109 | 0.109 | 0.029 |
| Residual Variance (σ²) | 0.070 | 0.069 | 0.032 |
| ICC (%) | 61.0 | 61.3 | 47.9 |

**Table S6.4.** Linear mixed-effects model for full-stimulus RMS broadband magnitude.

Three multilevel models were estimated: a non-interaction model, an interaction model including the SES–condition term, and an outlier-removed sensitivity model. The original dataset contained 140 observations across 70 clusters. For the outlier-removed model, 11 observations (7.9%) were identified as outliers, leaving 129 observations across 67 clusters. Intraclass correlation coefficients (ICCs) indicated that a substantial proportion of variance was attributable to between-cluster differences, with estimates of 59.7% for the non-interaction model, 60.0% for the interaction model, and 46.7% for the outlier-removed model, supporting the multilevel specification. The interaction between SES and condition was not statistically significant in the interaction model (p = .192), which was therefore retained for interpretation. To assess the robustness of the findings, we estimated an outlier-removed counterpart as a sensitivity model, which produced highly consistent results.

| **Parameter** | **Non-interaction Model [95% CI]** | **p-value** | **Interaction Model [95% CI]** | **p-value** | **Outlier-removed Model [95% CI]** | **p-value** |
| --- | --- | --- | --- | --- | --- | --- |
| Intercept | -2.315 [-2.812, -1.493] | <.001 | -2.343 [-2.845, -1.502] | <.001 | -1.522 [-1.758, -1.284] | <.001 |
| High-SES ᵃ | 0.195 [0.076, 0.302] | .002 | 0.250 [0.108, 0.387] | <.001 | 0.173 [0.101, 0.252] | <.001 |
| Babble ᵇ | -0.240 [-0.324, -0.153] | <.001 | -0.185 [-0.295, -0.054] | .008 | -0.224 [-0.284, -0.168] | <.001 |
| Working Memory | 0.028 [-0.006, 0.048] | .083 | 0.028 [-0.006, 0.048] | .083 | -0.006 [-0.015, 0.003] | .205 |
| High-SES ^a^ × Babble ^b^ |  |  | -0.110 [-0.288, 0.054] | .192 |  |  |

ᵃ Reference SES is Low-SES. ᵇ Reference condition is silence.

| **Component** | **Non-interaction Model** | **Interaction Model** | **Outlier-removed Model** |
| --- | --- | --- | --- |
| Cluster Variance (σ²) | 0.098 | 0.099 | 0.025 |
| Residual Variance (σ²) | 0.067 | 0.066 | 0.029 |
| ICC (%) | 59.7 | 60.0 | 46.7 |

**Supplementary Material 7.** Observed amplitude values of the fundamental frequency (F_0_), second harmonic (H_2_), and third harmonic (H_3_) by SES group and listening condition for a FFR elicited by a 170 ms /da/ syllable.

|  | **Low-SES** | |  | **High-SES** | |
| --- | --- | --- | --- | --- | --- |
|  | **Silence** | **Babble** |  | **Silence** | **Babble** |
| F_0_ | 0.067 (0.046; 0.099) | 0.051 (0.035; 0.100) |  | 0.087 (0.060; 0.139) | 0.085 (0.058; 0.107) |
| H_2_ | 0.036 (0.027; 0.049) | 0.019 (0.012; 0.027) |  | 0.046 (0.031; 0.061) | 0.028 (0.023; 0.040) |
| H_3_ | 0.019 (0.014; 0.028) | 0.014 (0.010; 0.020) |  | 0.022 (0.016; 0.028) | 0.014 (0.010; 0.021) |

Values are presented as the median amplitude in microvolts (µV), with the 25th and 75th percentiles shown in parentheses.

**Supplementary Material 8.** Details and comparison of linear mixed-effects models for frequency-specific (harmonic) magnitudes.

Three multilevel models were estimated: a non-interaction model, an interaction model including the SES–condition term, and an outlier-removed sensitivity model. The original dataset contained 420 observations across 70 clusters. For the outlier-removed model, 33 observations (7.9%) were identified as outliers, leaving 387 observations across the same 70 clusters. Intraclass correlation coefficients (ICCs) indicated that a substantial proportion of variance was attributable to between-cluster differences, with estimates of 55.5% for the non-interaction model, 55.4% for the interaction model, and 48.1% for the outlier-removed model, supporting the multilevel specification. The interaction between SES and condition was not statistically significant in the interaction model (p = .971), which was therefore retained for interpretation. To assess the robustness of the findings, we estimated an outlier-removed counterpart as a sensitivity model, which produced highly consistent results.

| **Parameter** | **Non-interaction Model [95% CI]** | **p-value** | **Interaction Model [95% CI]** | **p-value** | **Outlier-removed Model [95% CI]** | **p-value** |
| --- | --- | --- | --- | --- | --- | --- |
| Intercept | -3.570 [-4.283, -2.361] | <.001 | -3.571 [-4.288, -2.366] | <.001 | -3.529 [-4.208, -2.521] | <.001 |
| High-SES ᵃ | 0.265 [0.095, 0.426] | .003 | 0.266 [0.060, 0.458] | .014 | 0.287 [0.139, 0.428] | <.001 |
| Babble ᵇ | -0.406 [0.320, 0.489] | <.001 | 0.407 [0.265, 0.532] | <.001 | 0.378 [0.304, 0.459] | <.001 |
| H_2_ ^c^ | -0.871 [-1.003, -0.741] | <.001 | -0.871 [-1.003, -0.741] | <.001 | -0.765 [-0.911, -0.641] | <.001 |
| H_3_ ^c^ | -1.504 [-1.623, -1.383] | <.001 | -1.504 [-1.623, -1.383] | <.001 | -1.362 [-1.504, -1.248] | <.001 |
| Working Memory | 0.026 [-0.023, 0.054] | .167 | 0.026 [-0.023, 0.054] | .167 | 0.019 [-0.021, 0.046] | .250 |
| High-SES ^a^ × Babble ^b^ |  |  | -0.002 [-0.167, 0.173] | .971 |  |  |

ᵃ Reference SES is Low-SES. ᵇ Reference condition is silence. ^c^ Reference harmonic es fundamental frequency (F_0_). H_2_: second harmonic. H_3_: third harmonic.

| **Component** | **Non-interaction Model** | **Interaction Model** | **Outlier-removed Model** |
| --- | --- | --- | --- |
| Cluster Variance (σ²) | 0.237 | 0.237 | 0.165 |
| Residual Variance (σ²) | 0.190 | 0.191 | 0.177 |
| ICC (%) | 55.5 | 55.4 | 48.1 |

**Supplementary Material 9.** Observed z-score–transformed for stimulus-to-response correlation values.

|  | **Low-SES** | |  | **High-SES** | |
| --- | --- | --- | --- | --- | --- |
|  | **Silence** | **Babble** |  | **Silence** | **Babble** |
| Transition | 0.210 (0.185; 0.241) | 0.172 (0.158; 0194) |  | 0.218 (0.197; 0.230) | 0.209 (0.193; 0.224) |
| Steady-State | 0.182 (0.157; 0.206) | 0.132 (0.105; 0.157) |  | 0.202 (0.163; 0.234) | 0.176 (0.163; 0.198) |
| Full-stimulus | 0.160 (0.143; 0.177) | 0.133 (0.109; 0.152) |  | 0.170 (0.140; 0.194) | 0.157 (0.133; 0.177) |

Values are presented as z-score–transformed correlation coefficients, with the 25th and 75th percentiles shown in parentheses.

**Supplementary Material 10.** Details and comparison of linear mixed-effects models for z-score–transformed stimulus-to-response correlations.

**Table S10.1.** Transition stimulus-to-response correlation model comparison.

Three multilevel models were estimated: a non-interaction model, an interaction model including the SES–condition term, and an outlier-removed sensitivity model. The original dataset contained 140 observations across 70 clusters. For the outlier-removed model, 3 observations (2.1%) were identified as outliers, leaving 137 observations across the same 70 clusters. Intraclass correlation coefficients (ICCs) indicated that only a small proportion of variance was attributable to between-cluster differences, with estimates of 7.2% for the non-interaction model, 0.0% for the interaction model, and 11.5% for the outlier-removed model, supporting the multilevel specification. The interaction between SES and condition was statistically significant in the interaction model (p = .018) and remained significant in the outlier-removed model (p = .035); therefore, the interaction specification was retained for interpretation. To assess the robustness of the findings, we estimated an outlier-removed counterpart as a sensitivity model, which produced consistent results.

| **Parámetro** | **Non-interaction Model [95% CI]** | **p-value** | **Interaction Model [95% CI]** | **p-value** | **Outlier-removed Model [95% CI]** | **p-value** |
| --- | --- | --- | --- | --- | --- | --- |
| Intercept | 0.207 [0.196, 0.217] | <.001 | 0.209 [0.195, 0.221] | <.001 | 0.215 [0.201, 0.229] | <.001 |
| High-SES ^a^ | 0.018 [0.007, 0.030] | .001 | 0.006 [-0.009, 0.021] | .445 | 0.008 [-0.008, 0.025] | .330 |
| Babble ^b^ | -0.021 [-0.034, -0.008] | .003 | -0.033 [-0.052, -0.013] | .002 | -0.037 [-0.057, -0.017] | <.001 |
| High-SES ^a^ × Babble ^b^ | - | - | 0.026 [0.004, 0.047] | .018 | 0.026 [0.002, 0.051] | .035 |

ᵃ Reference SES is Low-SES. ᵇ Reference condition is silence.

| **Parámetro** | **Non-interaction Model** | **Interaction Model** | **Outlier-removed Model** |
| --- | --- | --- | --- |
| Varianza entre clusters (σ²) | <0.001 | <0.001 | <0.001 |
| Varianza residual (σ²) | 0.001 | 0.001 | 0.001 |
| ICC (%) | 7.2 | 0.0 | 11.5 |

**Table S10.2.** Steady-state stimulus-to-response correlation model comparison.

Two multilevel models were estimated: a non-interaction model and an interaction model including the SES–condition term. The original dataset contained 140 observations across 70 clusters. No outliers were identified; therefore, an outlier-removed specification was not estimated. Intraclass correlation coefficients (ICCs) indicated that a modest proportion of variance was attributable to between-cluster differences, with estimates of 20.2% for the non-interaction model and 22.5% for the interaction model, supporting the multilevel specification. The interaction between SES and condition was statistically significant in the interaction model (p = .034), which was therefore retained for interpretation.

| **Parameter** | **Non-interaction Model [95% CI]** | **p-value** | **Interaction Model [95% CI]** | **p-value** |
| --- | --- | --- | --- | --- |
| Intercept | 0.148 [0.122, 0.177] | <.001 | 0.154 [0.127, 0.183] | <.001 |
| High-SES ᵃ | 0.029 [0.017, 0.041] | <.001 | 0.017 [-0.001, 0.035] | .065 |
| Babble ᵇ | -0.034 [-0.046, -0.024] | <.001 | -0.046 [-0.064, -0.030] | <.001 |
| Working Memory | 0.001 [0.000, 0.002] | .029 | 0.001 [0.000, 0.002] | .029 |
| High-SES ^a^ × Babble ^b^ |  |  | 0.024 [0.002, 0.046] | .034 |

ᵃ Reference SES is Low-SES. ᵇ Reference condition is silence.

| **Component** | **Non-interaction Model** | **Interaction Model** |
| --- | --- | --- |
| Cluster Variance (σ²) | 0.00030 | 0.00033 |
| Residual Variance (σ²) | 0.00119 | 0.00114 |
| ICC (%) | 20.2 | 22.5 |

**Table S10.3.** Full-stimulus-to-response correlation model comparison.

Three multilevel models were estimated: a non-interaction model, an interaction model including the SES × condition term, and an outlier-removed model. Of the original 140 observations across 70 clusters, 2 outliers (1.4%) were identified with the IQR method (k = 1.5) applied within SES–condition groups, leaving 138 observations across the same 70 clusters for the outlier-removed model. Intraclass correlation coefficients (ICCs) indicated that a modest proportion of the variance was attributable to between-cluster differences, with estimates of 20.0% for the non-interaction model, 22.2% for the interaction model, and 24.1% for the outlier-removed model, supporting the multilevel specification. The SES × condition interaction was statistically significant (p = .041 in the interaction model; p = .030 in the outlier-removed model). Accordingly, the interaction specification was retained for interpretation, and the sensitivity analysis confirmed the robustness of the findings.

| **Parameter** | **Non-interaction Model [95% CI]** | **p-value** | **Interaction Model [95% CI]** | **p-value** | **Outlier-removed Model [95% CI]** | **p-value** |
| --- | --- | --- | --- | --- | --- | --- |
| Intercept | 0.127 [0.108, 0.150] | <.001 | 0.132 [0.112, 0.155] | <.001 | 0.136 [0.116, 0.160] | <.001 |
| High-SES ᵃ | 0.012 [0.002, 0.021] | .014 | 0.002 [-0.010, 0.015] | .732 | 0.003 [-0.009, 0.015] | .661 |
| Babble ᵇ | -0.020 [-0.029, -0.010] | <.001 | -0.029 [-0.044, -0.015] | <.001 | -0.029 [-0.043, -0.017] | <.001 |
| Working Memory | 0.001 [0.000, 0.002] | .008 | 0.001 [0.000, 0.002] | .008 | 0.001 [0.000, 0.002] | .027 |
| High-SES ^a^ × Babble ^b^ | - | - | 0.019 [0.001, 0.038] | .041 | 0.019 [0.002, 0.037] | .030 |

ᵃ Reference SES is Low-SES. ᵇ Reference condition is silence.

| **Component** | **Non-interaction Model** | **Interaction Model** | **Outlier-removed Model** |
| --- | --- | --- | --- |
| Varianza entre clusters (σ²) | <0.001 | <0.001 | <0.001 |
| Varianza residual (σ²) | 0.001 | 0.001 | 0.001 |
| ICC (%) | 18.3 | 20.4 | 22.7 |

**Supplementary Material 11.** Observed z-score–transformed for response-to-response correlation values.

|  | **Low-SES** | |  | **High-SES** | |
| --- | --- | --- | --- | --- | --- |
|  | **Silence** | **Babble** |  | **Silence** | **Babble** |
| Transition | 0.961 [0.576; 1.222] | 0.358 [0.162; 0.513] |  | 0.923 [0.700; 1.142] | 0.397 [0.233; 0.702] |
| Steady-State | 0.855 [0.671; 1.045] | 0.464; [0.240; 0.825] |  | 0.857 [0.558; 1.194] | 0.616 [0.453; 0.732] |
| Full-stimulus | 0.818 [0.594; 1.047] | 0.408 [0.201; 0.730] |  | 0.811 [0.599; 1.201] | 0.508 [0.368; 0.693] |

Values are presented as z-score–transformed correlation coefficients, with the 25th and 75th percentiles shown in parentheses.

**Supplementary Material 12.** Details and comparison of linear mixed-effects models for z-score–transformed response-to-response correlations.

**Table S12.1.** Transition response-to-response correlation model comparison.

Three multilevel models were estimated: a non-interaction model, an interaction model including the SES–condition term, and an outlier-removed sensitivity model. The original dataset contained 140 observations across 70 clusters. For the outlier-removed model, 10 observations (7.1%) were identified as outliers, leaving 130 observations across 67 clusters. Intraclass correlation coefficients (ICCs) indicated that a substantial proportion of variance was attributable to between-cluster differences, with estimates of 64.6% for the non-interaction model, 64.2% for the interaction model, and 42.7% for the outlier-removed model, supporting the multilevel specification. The interaction between SES and condition was not statistically significant in the interaction model (p = 1.000), which was therefore retained for interpretation. To assess the robustness of the findings, we estimated an outlier-removed counterpart as a sensitivity model, which produced highly consistent results.

| **Parameter** | **Non-interaction Model [95% CI]** | **p-value** | **Interaction Model [95% CI]** | **p-value** | **Outlier-removed Model [95% CI]** | **p-value** |
| --- | --- | --- | --- | --- | --- | --- |
| Intercept | 0.924 [0.818, 1.028] | <0.001 | 0.924 [0.808, 1.042] | <0.001 | 0.901 [0.803, 1.007] | <0.001 |
| High-SES ^a^ | 0.080 [-0.058, 0.222] | 0.255 | 0.080 [-0.093, 0.252] | 0.356 | 0.005 [-0.108, 0.109] | 0.993 |
| Babble ^b^ | -0.504 [-0.588, -0.422] | <0.001 | -0.504 [-0.627, -0.385] | <0.001 | -0.520 [-0.605, -0.428] | <0.001 |
| High-SES ^a^ × Babble ^b^ | - | - | -0.000 [-0.165, 0.168] | 1.000 | - | - |

ᵃ Reference SES is Low-SES. ᵇ Reference condition is silence.

| **Component** | **Non-interaction Model** | **Interaction Model** | **Outlier-removed Model** |
| --- | --- | --- | --- |
| Varianza entre clusters (σ²) | 0.117 | 0.116 | 0.049 |
| Varianza residual (σ²) | 0.064 | 0.065 | 0.066 |
| ICC (%) | 64.6 | 64.2 | 42.7 |

**Table S12.2.** Steady-state response-to-response correlation model comparison.

Three multilevel models were estimated: a non-interaction model, an interaction model including the SES–condition term, and an outlier-removed sensitivity model. The original dataset contained 140 observations across 70 clusters. For the outlier-removed model, 7 observations (5.0%) were identified as outliers, leaving 133 observations across the same 70 clusters. Intraclass correlation coefficients (ICCs) indicated that a substantial proportion of variance was attributable to between-cluster differences, with estimates of 73.6% for the non-interaction model, 73.8% for the interaction model, and 63.7% for the outlier-removed model, supporting the multilevel specification. The interaction between SES and condition was not statistically significant in the interaction model (p = .190), which was therefore retained for interpretation. To assess the robustness of the findings, we estimated an outlier-removed counterpart as a sensitivity model, which produced highly consistent results.

| **Parameter** | **Non-interaction Model [95% CI]** | **p-value** | **Interaction Model [95% CI]** | **p-value** | **Outlier-removed Model [95% CI]** | **p-value** |
| --- | --- | --- | --- | --- | --- | --- |
| Intercept | 0.843 [0.747, 0.936] | <0.001 | 0.868 [0.770, 0.964] | <0.001 | 0.844 [0.750, 0.925] | <0.001 |
| High-SES ^a^ | 0.132 [-0.017, 0.282] | 0.083 | 0.082 [-0.078, 0.242] | 0.307 | 0.112 [-0.010, 0.255] | 0.068 |
| Babble ^b^ | -0.266 [-0.342, -0.191] | <0.001 | -0.315 [-0.435, -0.200] | <0.001 | -0.309 [-0.374, -0.215] | <0.001 |
| High-SES ^a^ × Babble ^b^ | - | - | 0.099 [-0.051, 0.248] | 0.190 | - | - |

ᵃ Reference SES is Low-SES. ᵇ Reference condition is silence.

| **Component** | **Non-interaction Model** | **Interaction Model** | **Outlier-removed Model** |
| --- | --- | --- | --- |
| Cluster Variance (σ²) | 0.144 | 0.144 | 0.095 |
| Residual Variance (σ²) | 0.052 | 0.051 | 0.054 |
| ICC (%) | 73.6 | 73.8 | 63.7 |

**Table S12.3.** Full-stimulus response-to-response correlation model comparison.

Three multilevel models were estimated: a non-interaction model, an interaction model including the SES–condition term, and an outlier-removed sensitivity model. The original dataset contained 140 observations across 70 clusters. For the outlier-removed model, 5 observations (3.6%) were identified as outliers, leaving 135 observations across the same 70 clusters. Intraclass correlation coefficients (ICCs) indicated that a large proportion of variance was attributable to between-cluster differences, with estimates of 77.1% for the non-interaction model, 76.9% for the interaction model, and 70.1% for the outlier-removed model, supporting the multilevel specification. The interaction between SES and condition was not statistically significant in the interaction model (p = .593), which was therefore retained for interpretation. To assess the robustness of the findings, we estimated an outlier-removed counterpart as a sensitivity model, which produced highly consistent results.

| **Parameter** | **Non-interaction Model [95% CI]** | **p-value** | **Interaction Model [95% CI]** | **p-value** | **Outlier-removed Model [95% CI]** | **p-value** |
| --- | --- | --- | --- | --- | --- | --- |
| Intercept | 0.833 [0.741, 0.923] | <0.001 | 0.843 [0.749, 0.937] | <0.001 | 0.842 [0.749, 0.925] | <0.001 |
| High-SES ^a^ | 0.117 [-0.022, 0.259] | 0.103 | 0.098 [-0.052, 0.251] | 0.196 | 0.100 [-0.022, 0.238] | 0.103 |
| Babble ^b^ | -0.323 [-0.388, -0.260] | <0.001 | -0.341 [-0.445, -0.244] | <0.001 | -0.359 [-0.417, -0.279] | <0.001 |
| High-SES ^a^ × Babble ^b^ | - | - | 0.037 [-0.094, 0.168] | 0.593 | - | - |

ᵃ Reference SES is Low-SES. ᵇ Reference condition is silence.

| **Component** | **Non-interaction Model** | **Interaction Model** | **Outlier-removed Model** |
| --- | --- | --- | --- |
| Cluster Variance (σ²) | 0.131 | 0.131 | 0.098 |
| Residual Variance (σ²) | 0.039 | 0.039 | 0.042 |
| ICC (%) | 77.1 | 76.9 | 70.1 |

**Supplementary Material 13.** Observed speech recognition threshold (SRT) and Speech, Spatial and Qualities of Hearing Scale (SSQ12) values by SES group.

| **Measure** |  | **Low-SES** |  | **High-SES** |
| --- | --- | --- | --- | --- |
| SRT (Behavioral SPiN Performance) |  | -1.800 [-3.725 , -0.675] |  | -2.350 [-3.300 , -1.400] |
| SSQ12 (Self-reported SPiN Performance) |  | 7.558 [6.589 , 8.277] |  | 7.783 [6.440 , 8.368] |

Values represent the median with the 25th and 75th percentiles shown in parentheses. SRT: Defined as the lowest signal-to-noise ratio (SNR) at which the listener correctly recognizes at least 50% of the presented monosyllabic words. SSQ12: Self-perceived listening ability in everyday situations, measured using the validated Spanish SSQ12 (scores from 0 = great difficulty to 10 = no difficulty).

**Supplementary Material 14.** Details and comparison of linear models for behavioral SPiN performance (SRT).

**Table S14.1.** Behavioral SPiN performance (SRT) model comparison using the onset-latency contrast (p09).

Three multilevel models were estimated: (1) a SES-only model, (2) an additive model including the FFR onset-latency contrast, and (3) an interaction model testing whether the association between SES and behavioral SPiN performance varied as a function of the FFR onset predictor. The original dataset included 70 subjects. In the outlier-removed sensitivity analysis, 2 observations (2.9%) were excluded, yielding a final sample of 68 subjects. The interaction term was not statistically significant in either the original dataset (p = .283) or the outlier-removed dataset (p = .806); therefore, the additive specification was retained for interpretation. In the SES-only model, SES was not significant in the full dataset (p = .149) but reached statistical significance after outlier removal (p = .042). In the additive model, SES was not significant in either the full dataset (p = .561) or the outlier-removed dataset (p = .157), whereas the FFR onset-latency contrast was significant in both datasets, with p = .008 in the full sample and p = .015 after outlier removal.

| **Parameter** | **SES-only Model [95% CI]** | **p-value** | **SES + FFR Contrast** ^b^ **Model [95% CI]** | **p-value** | **Interaction Model [95% CI]** | **p-value** |
| --- | --- | --- | --- | --- | --- | --- |
| **Original Dataset** |  |  |  |  |  |  |
| Intercept | -1.911 [-2.492, -1.315] | < .001 | -3.244 [-4.360, -2.310] | < .001 | -2.670 [-3.939, -1.509] | < .001 |
| High-SES ᵃ | −0.725 [-1.663, 0.251] | .149 | -0.293 [-1.196, 0.716] | .561 | -1.180 [-2.816, 0.377] | .148 |
| FFR Contrast ^b^ | - | - | 1.039 [0.378, 1.838] | .008 | 0.592 [-0.336, 1.637] | .209 |
| High-SES ᵃ× FFR Contrast ^b^ | - | - | - | - | 0.808 [-0.601, 2.364] | .283 |
|  |  |  |  |  |  |  |
| **Outlier-removed Dataset** |  |  |  |  |  |  |
| Intercept | -1.911 [-2.481, -1.315] | < .001 | -2.762 [-3.560, -2.022] | < .001 | -2.670 [-3.947, -1.543] | < .001 |
| High-SES ᵃ | -0.812 [-1.551, -0.069] | .042 | -0.548 [-1.294, 0.169] | .157 | -0.697 [-2.023, 0.671] | .286 |
| FFR Contrast ^b^ | - | - | 0.663 [0.150, 1.228] | .015 | 0.592 [-0.338, 1.622] | .209 |
| High-SES ᵃ × FFR Contrast ^b^ | - | - | - | - | 0.136 [-1.025, 1.253] | .806 |

ᵃ Reference SES is Low-SES. ᵇ In this model, the FFR contrast refers to the latency difference of the p09 onset component between the babble condition and the silent condition.

**Table S14.2.** Behavioral SPiN performance model comparison using the transition-latency contrast (p23, p32, p42, and p52).

Three multilevel models were estimated: (1) a SES-only model, (2) an additive model including the FFR transition-latency contrast, and (3) an interaction model testing whether the association between SES and behavioral SPiN performance varied as a function of the FFR transition-latency predictor. The original dataset included 70 subjects. In the outlier-removed sensitivity analysis, 2 observations (2.9%) were excluded, yielding a final sample of 68 subjects. The interaction term was not statistically significant in the original dataset (p = .646) or in the outlier-removed model (p = .264); therefore, the additive specification was retained for interpretation. In the SES-only model, SES was not significant in the full dataset (p = .069) but reached statistical significance after outlier removal (p = .014). In the additive model, SES remained significant in the outlier-removed dataset (p = .012), whereas the FFR transition-latency contrast was not significant in either dataset, with p = .883 in the full sample and p = .569 after outlier removal.

| **Parameter** | **SES-only Model [95% CI]** | **p-value** | **SES + FFR Contrast** ^b^ **Model [95% CI]** | **p-value** | **Interaction Model [95% CI]** | **p-value** |
| --- | --- | --- | --- | --- | --- | --- |
| **Original Dataset** |  |  |  |  |  |  |
| Intercept | -1.936 [-2.832, -1.050] | < .001 | -1.923 [-2.851, -1.013] | < .001 | -1.970 [-2.888, -1.049] | < .001 |
| High-SES ᵃ | -0.454 [-0.936, 0.028] | .069 | -0.459 [-0.940, 0.036] | .070 | -0.357 [-1.013, 0.293] | .287 |
| Peak | 0.000 [-0.022, 0.023] | .993 | 0.000 [-0.022, 0.023] | .987 | 0.000 [-0.022, 0.023] | .980 |
| FFR Contrast ^b^ | - | - | -0.018 [-0.259, 0.227] | .883 | 0.021 [-0.305, 0.355] | .901 |
| High-SES ᵃ× FFR Contrast ^b^ | - | - | - | - | -0.112 [-0.580, 0.376] | .646 |
|  |  |  |  |  |  |  |
| **Outlier-removed Dataset** |  |  |  |  |  |  |
| Intercept | -1.936 [-2.695, -1.164] | < .001 | -1.886 [-2.678, -1.078] | < .001 | -1.991 [-2.803, -1.166] | < .001 |
| High-SES ᵃ | -0.518 [-0.925, -0.108] | .014 | -0.538 [-0.956, -0.126] | .012 | -0.305 [-0.885, 0.278] | .314 |
| Peak | 0.000 [-0.019, 0.019] | 1.000 | 0.001 [-0.018, 0.020] | .945 | 0.001 [-0.018, 0.020] | .930 |
| FFR Contrast ^b^ | - | - | -0.069 [-0.307, 0.164] | .569 | 0.020 [-0.303, 0.357] | .904 |
| High-SES ᵃ× FFR Contrast ^b^ | - | - | - | - | -0.256 [-0.717, 0.175] | .264 |

ᵃ Reference SES is Low-SES. ^b^ In this model, the FFR contrast refers to the latency difference of the transition components (p23, p32, p42, and p52) between the babble condition and the silent condition.

**Table S14.3.** Behavioral SPiN performance model comparison using the transition stimulus-to-response correlation contrast.

Three multilevel models were estimated: (1) a SES-only model, (2) an additive model including the FFR transition correlation contrast, and (3) an interaction model testing whether the association between SES and behavioral SPiN performance varied as a function of the FFR transition-correlation predictor. The original dataset included 70 subjects. In the outlier-removed sensitivity analysis, 2 observations (2.9%) were excluded, yielding a final sample of 68 subjects. The interaction term was not statistically significant in either the original dataset (p = .087) or the outlier-removed dataset (p = .128); therefore, the additive specification was retained for interpretation. In the SES-only model, SES was not significant in the full dataset (p = .373) nor in the outlier-removed dataset (p = .228). In the additive model, SES remained non-significant in both the full dataset (p = .611) and the outlier-removed dataset (p = .485), whereas the FFR transition-correlation contrast was not significant in the full dataset (p = .082) but reached statistical significance after outlier removal (p = .045).

| **Parameter** | **SES-only Model [95% CI]** | **p-value** | **SES + FFR Contrast** ^b^ **Model [95% CI]** | **p-value** | **Interaction Model [95% CI]** | **p-value** |
| --- | --- | --- | --- | --- | --- | --- |
| **Original Dataset** |  |  |  |  |  |  |
| Intercept | -1.936 [-2.564, -1.302] | < .001 | -2.147 [-2.760, -1.476] | < .001 | -2.278 [-2.913, -1.627] | < .001 |
| High-SES ᵃ | -0.454 [-1.402, 0.539] | .373 | -0.266 [-1.285, 0.730] | .611 | -0.095 [-1.060, 0.920] | .849 |
| FFR Contrast ^b^ | - | - | 6.725 [-1.618, 13.614] | .082 | 10.872 [1.775, 19.255] | .010 |
| High-SES ᵃ× FFR Contrast ^b^ | - | - | - | - | -15.804 [-38.166, 0.883] | .087 |
|  |  |  |  |  |  |  |
| **Outlier-removed Dataset** |  |  |  |  |  |  |
| Intercept | -1.936 [-2.568, -1.309] | < .001 | -2.172 [-2.789, -1.515] | < .001 | -2.278 [-2.904, -1.641] | < .001 |
| High-SES ᵃ | -0.518 [-1.322, 0.295] | .228 | -0.304 [-1.143, 0.516] | .485 | -0.171 [-0.973, 0.657] | .686 |
| FFR Contrast ^b^ | - | - | 7.518 [-0.340, 14.536] | .045 | 10.872 [1.938, 19.320] | .010 |
| High-SES ᵃ× FFR Contrast ^b^ | - | - | - | - | -12.884 [-31.989, 3.138] | .128 |

ᵃ Reference SES is Low-SES. ᵇ In this model, the FFR contrast refers to the transition stimulus-to-response correlation difference between the silent condition and the babble condition.

**Table S14.4.** Behavioral SPiN performance model comparison using the steady-state stimulus–to–response correlation contrast.

Three multilevel models were estimated: (1) a SES-only model, (2) an additive model including the FFR steady-state correlation contrast, and (3) an interaction model testing whether the association between SES and behavioral SPiN performance varied as a function of the FFR steady-state predictor. The original dataset included 70 subjects. In the outlier-removed sensitivity analysis, 2 observations (2.9%) were excluded, yielding a final sample of 68 subjects. The interaction term was not statistically significant in either the original dataset (p = .516) or the outlier-removed dataset (p = .622); therefore, the additive specification was retained for interpretation. In the SES-only model, SES was not significant in the full dataset (p = .373) nor in the outlier-removed dataset (p = .228). In the additive model, SES remained non-significant in both the full dataset (p = .650) and the outlier-removed dataset (p = .519), whereas the FFR steady-state correlation contrast was marginally non-significant in the full dataset (p = .053) but reached statistical significance after outlier removal (p = .029).

| **Parameter** | **SES-only Model [95% CI]** | **p-value** | **SES + FFR Contrast** ^b^ **Model [95% CI]** | **p-value** | **Interaction Model [95% CI]** | **p-value** |
| --- | --- | --- | --- | --- | --- | --- |
| **Original Dataset** |  |  |  |  |  |  |
| Intercept | -1.936 [-2.564, -1.302] | < .001 | -2.360 [-3.061, -1.576] | < .001 | -2.486 [-3.220, -1.642] | < .001 |
| High-SES ᵃ | -0.454 [-1.402, 0.539] | .373 | -0.232 [-1.196, 0.734] | .650 | -0.013 [-1.169, 1.169] | .983 |
| FFR Contrast ^b^ | - | - | 9.381 [0.102, 18.895] | .053 | 12.166 [0.655, 25.221] | .031 |
| High-SES ᵃ× FFR Contrast ^b^ | - | - | - | - | -7.121 [-28.384, 12.891] | .516 |
|  |  |  |  |  |  |  |
| **Outlier-removed Dataset** |  |  |  |  |  |  |
| Intercept | -1.936 [-2.568, -1.309] | < .001 | -2.395 [-3.095, -1.623] | < .001 | -2.486 [-3.220, -1.653] | < .001 |
| High-SESᵃ | -0.518 [-1.322, 0.295] | .228 | -0.288 [-1.147, 0.549] | .519 | -0.127 [-1.096, 0.770] | .795 |
| FFR Contrast ^b^ | - | - | 10.169 [1.276, 19.336] | .029 | 12.166 [0.907, 24.784] | .031 |
| High-SES ᵃ× FFR Contrast ^b^ | - | - | - | - | -5.145 [-24.446, 14.675] | .622 |

ᵃ Reference SES is Low-SES. ᵇ In this model, the FFR contrast refers to the steady-state stimulus-to-response correlation difference between the silent condition and the babble condition.

**Table S14.5.** Behavioral SPiN performance model comparison using the full-stimulus stimulus–to–response correlation contrast.

Three multilevel models were estimated: (1) a SES-only model, (2) an additive model including the FFR full-stimulus correlation contrast, and (3) an interaction model testing whether the association between SES and behavioral SPiN performance varied as a function of the FFR full-stimulus predictor. The original dataset included 70 subjects. In the outlier-removed sensitivity analysis, 2 observations (2.9%) were excluded, yielding a final sample of 68 subjects. The interaction term was not statistically significant in either the original dataset (p = .214) or the outlier-removed dataset (p = .291); therefore, the additive specification was retained for interpretation. In the SES-only model, SES was not significant in the full dataset (p = .373) nor in the outlier-removed dataset (p = .228). In the additive model, SES remained non-significant in both the full dataset (p = .721) and the outlier-removed dataset (p = .618), whereas the FFR full-stimulus correlation contrast was statistically significant in both the full dataset (p = .018) and the outlier-removed dataset (p < .001).

| **Parameter** | **SES-only Model [95% CI]** | **p-value** | **SES + FFR Contrast** ^b^ **Model [95% CI]** | **p-value** | **Interaction Model [95% CI]** | **p-value** |
| --- | --- | --- | --- | --- | --- | --- |
| **Original Dataset** |  |  |  |  |  |  |
| Intercept | -1.936 [-2.564, -1.302] | < .001 | -2.376 [-2.991, -1.675] | < .001 | -2.595 [-3.235, -1.997] | < .001 |
| High-SES ᵃ | -0.454 [-1.402, 0.539] | .373 | -0.165 [-1.048, 0.704] | .721 | 0.173 [-0.852, 1.293] | .765 |
| FFR Contrast ^b^ | - | - | 15.369 [2.110, 27.467] | .018 | 23.008 [11.951, 40.248] | .003 |
| High-SES ᵃ× FFR Contrast ^b^ | - | - | - | - | -19.793 [-55.966, 4.509] | .214 |
|  |  |  |  |  |  |  |
| **Outlier-removed Dataset** |  |  |  |  |  |  |
| Intercept | -1.936 [-2.568, -1.309] | < .001 | -2.475 [-3.051, -1.882] | < .001 | -2.595 [-3.217, -1.992] | < .001 |
| High-SESᵃ | -0.518 [-1.322, 0.295] | .228 | -0.199 [-0.973, 0.553] | .618 | 0.004 [-0.766, 0.815] | .992 |
| FFR Contrast ^b^ | - | - | 18.806 [10.233, 29.135] | < .001 | 23.008 [11.950, 39.983] | .003 |
| High-SES ᵃ× FFR Contrast ^b^ | - | - | - | - | -11.357 [-34.696, 5.679] | .291 |

ᵃ Reference SES is Low-SES. ^b^ In this model, the FFR contrast refers to the full-stimulus stimulus-to-response correlation difference between the silent condition and the babble condition.

**Supplementary Material 15.** Details and comparison of linear models for self-reported SPiN performance (SSQ-12).

**Table S15.1.** Self-reported SPiN performance (SSQ-12) model comparison using the onset-latency contrast (p09).

Three multilevel models were estimated: (1) a SES-only model, (2) an additive model including the FFR onset-latency contrast, and (3) an interaction model testing whether the association between SES and self-reported SPiN performance varied as a function of the FFR onset predictor. The original dataset included 65 subjects. No sensitivity analysis was conducted for this outcome because no outliers were identified. The interaction term was not statistically significant (p = .675); therefore, the additive specification was retained for interpretation. In the SES-only model, SES was not significant (p = .892). In the additive model, SES remained non-significant (p = .715), and the FFR onset-latency contrast was also non-significant (p = .109).

| **Parameter** | **SES-only Model [95% CI]** | **p-value** | **SES + FFR Contrast** ^b^ **Model [95% CI]** | **p-value** | **Interaction Model [95% CI]** | **p-value** |
| --- | --- | --- | --- | --- | --- | --- |
| **Original Dataset** |  |  |  |  |  |  |
| Intercept | 7.481 [7.019, 7.921] | < .001 | 6.997 [6.220, 7.710] | < .001 | 7.139 [5.982, 8.107] | < .001 |
| High-SESᵃ | -0.043 [-0.644, 0.573] | .892 | 0.114 [-0.468, 0.707] | .715 | -0.106 [-1.393, 1.254] | .875 |
| FFR Contrast ^b^ | - | - | 0.377 [-0.057, 0.858] | .109 | 0.267 [-0.354, 1.088] | .431 |
| High-SES ᵃ× FFR Contrast ^b^ | - | - | - | - | 0.200 [-0.786, 1.142] | .675 |

ᵃ Reference SES is Low-SES. ᵇ In this model, the FFR contrast refers to the latency difference of the p09 onset component between the babble condition and the silent condition.

**Table S15.2.** Self-reported SPiN performance (SSQ-12) model comparison using the transition-latency contrast (p23, p32, p42, and p52).

Three multilevel models were estimated: (1) a SES-only model, (2) an additive model including the FFR transition-latency contrast, and (3) an interaction model testing whether the association between SES and self-reported SPiN performance varied as a function of the FFR transition predictor. The original dataset included 70 subjects. No outliers were identified for this outcome; therefore, no outlier-removed sensitivity analysis was conducted. The interaction term was not statistically significant (p = .576); therefore, the additive specification was retained for interpretation. In the SES-only model, SES was not significant (p = .891). In the additive model, SES remained non-significant (p = .949), and the FFR transition-latency contrast was also non-significant (p = .670).

| **Parameter** | **SES-only Model [95% CI]** | **p-value** | **SES + FFR Contrast** ^b^ **Model [95% CI]** | **p-value** | **Interaction Model [95% CI]** | **p-value** |
| --- | --- | --- | --- | --- | --- | --- |
| **Original Dataset** |  |  |  |  |  |  |
| Intercept | 7.491 [6.944, 8.025] | < .001 | 7.517 [6.990, 8.041] | < .001 | 7.478 [6.904, 8.034] | < .001 |
| High-SESᵃ | 0.020 [-0.263, 0.298] | .891 | 0.010 [-0.281, 0.303] | .949 | 0.094 [-0.332, 0.520] | .674 |
| Peak | 0.000 [-0.013, 0.013] | 1.000 | 0.000 [-0.013, 0.014] | .958 | 0.001 [-0.013, 0.014] | .948 |
| FFR Contrast ^b^ | - | - | -0.037 [-0.204, 0.128] | .670 | -0.004 [-0.229, 0.214] | .973 |
| High-SES ᵃ× FFR Contrast ^b^ | - | - | - | - | -0.093 [-0.412, 0.225] | .576 |

ᵃ Reference SES is Low-SES. ^b^ In this model, the FFR contrast refers to the latency difference of the transition components (p23, p32, p42, and p52) between the babble condition and the silent condition.

**Table S15.3.** Self-reported SPiN performance (SSQ-12) model comparison using the transition stimulus-to-response correlation contrast.

Three multilevel models were estimated: (1) a SES-only model, (2) an additive model including the FFR transition correlation contrast, and (3) an interaction model testing whether the association between SES and self-reported SPiN performance varied as a function of the FFR transition-correlation predictor. The original dataset included 70 subjects. No outliers were identified for this outcome; therefore, no outlier-removed sensitivity analysis was conducted. The interaction term was not statistically significant (p = .591); therefore, the additive specification was retained for interpretation. In the SES-only model, SES was not significant (p = .946). In the additive model, SES remained non-significant (p = .601), and the FFR transition-correlation contrast was also non-significant (p = .118).

| **Parameter** | **SES-only Model [95% CI]** | **p-value** | **SES + FFR Contrast ᵇ Model [95% CI]** | **p-value** | **Interaction Model [95% CI]** | **p-value** |
| --- | --- | --- | --- | --- | --- | --- |
| **Original Dataset** |  |  |  |  |  |  |
| Intercept | 7.491 [7.050, 7.913] | < .001 | 7.342 [6.899, 7.728] | < .001 | 7.375 [6.939, 7.740] | < .001 |
| High-SES ᵃ | 0.020 [-0.556, 0.597] | .946 | 0.153 [-0.397, 0.732] | .601 | 0.109 [-0.404, 0.692] | .703 |
| FFR Contrast ᵇ | - | - | 4.747 [-1.146, 10.868] | .118 | 3.688 [-3.219, 10.920] | .280 |
| High-SES ᵃ × FFR Contrast ᵇ | - | - | - | - | 4.034 [-10.750, 16.347] | .591 |

ᵃ Reference SES is Low-SES. ᵇ In this model, the FFR contrast refers to the transition stimulus-to-response correlation difference between the silent condition and the babble condition.

**Table S15.4.** Self-reported SPiN performance (SSQ-12) model comparison using the steady-state stimulus-to-response correlation contrast.

Three multilevel models were estimated: (1) a SES-only model, (2) an additive model including the FFR steady-state correlation contrast, and (3) an interaction model testing whether the association between SES and self-reported SPiN performance varied as a function of the FFR steady-state predictor. The original dataset included 70 subjects. No outliers were identified for this outcome; therefore, no outlier-removed sensitivity analysis was conducted. The interaction term was not statistically significant (p = .326); therefore, the additive specification was retained for interpretation. In the SES-only model, SES was not significant (p = .946). In the additive model, SES remained non-significant (p = .976), and the FFR steady-state correlation contrast was also non-significant (p = .896).

| **Parameter** | **SES-only Model [95% CI]** | **p-value** | **SES + FFR Contrast** ᵇ **Model [95% CI]** | **p-value** | **Interaction Model [95% CI]** | **p-value** |
| --- | --- | --- | --- | --- | --- | --- |
| Intercept | 7.491 [7.050, 7.913] | < .001 | 7.512 [7.018, 8.024] | < .001 | 7.396 [6.934, 7.970] | < .001 |
| High-SES ᵃ | 0.020 [-0.556, 0.597] | .946 | 0.009 [-0.564, 0.596] | .976 | 0.211 [-0.548, 0.842] | .570 |
| FFR Contrast ᵇ | - | - | -0.462 [-7.297, 5.242] | .896 | 2.109 [-7.969, 7.852] | .608 |
| High-SES ᵃ × FFR Contrast ᵇ | - | - | - | - | -6.574 [-16.865, 9.231] | .326 |

ᵃ Reference SES is Low-SES. ᵇ In this model, the FFR contrast refers to the steady-state stimulus-to-response correlation difference between the silent condition and the babble condition.

**Table S15.5.** Self-reported SPiN performance (SSQ-12) model comparison using the full-stimulus stimulus–to–response correlation contrast.

Three multilevel models were estimated: (1) a SES-only model, (2) an additive model including the FFR full-stimulus correlation contrast, and (3) an interaction model testing whether the association between SES and self-reported SPiN performance varied as a function of the FFR full-stimulus predictor. The original dataset included 70 subjects. No outliers were identified for this outcome; therefore, no outlier-removed sensitivity analysis was conducted. The interaction term was not statistically significant (p = .472); therefore, the additive specification was retained for interpretation. In the SES-only model, SES was not significant (p = .946). In the additive model, SES remained non-significant (p = .702), and the FFR full-stimulus correlation contrast was also non-significant (p = .156).

| **Parameter** | **SES-only Model [95% CI]** | **p-value** | **SES + FFR Contrast** ᵇ **Model [95% CI]** | **p-value** | **Interaction Model [95% CI]** | **p-value** |
| --- | --- | --- | --- | --- | --- | --- |
| Intercept | 7.491 [7.050, 7.913] | < .001 | 7.349 [6.865, 7.790] | < .001 | 7.409 [6.918, 7.901] | < .001 |
| High-SES ᵃ | 0.020 [-0.556, 0.597] | .946 | 0.113 [-0.454, 0.698] | .702 | 0.021 [-0.620, 0.646] | .948 |
| FFR Contrast ᵇ | - | - | 4.949 [-2.222, 11.195] | .156 | 2.873 [-8.937, 11.288] | .567 |
| High-SES ᵃ × FFR Contrast ᵇ | - | - | - | - | 5.380 [-7.472, 20.901] | .472 |

ᵃ Reference SES is Low-SES. ^b^ In this model, the FFR contrast refers to the full-stimulus stimulus–to–response correlation difference between the silent condition and the babble condition.
